# Eco-friendly corrosion inhibitor chitosan methionine for carbon steel in 1 M hydrochloric acid solution: experimental and theoretical approach

**DOI:** 10.1038/s41598-025-98981-2

**Published:** 2025-05-07

**Authors:** S. F. Hamza, Seham Shahen, Amal M. Abdel‑karim, Ahmed A. El-Rashedy, Amira M. Hyba

**Affiliations:** 1 Department of Chemistry, Faculty of Science (Girls), Al-Azhar University, Yousef Abbas St, P.O. Box: 11754, Nasr City, Cairo Egypt; 2 Physical Chemistry Department, National Research Centre, 33 El Bohouth St. Dokki, P.O.12622, Giza, Egypt; 3 Natural and Microbial Products Department, National Research Centre, 33 El Bohouth St. Dokki, P.O.12622, Giza, Egypt

**Keywords:** Chitosan derivative, Green corrosion inhibitor, Carbon steel, Electrochemical measurements, Theoretical calculations, Chemistry, Engineering

## Abstract

There is a high demand for high performance, effective and eco-friendly corrosion inhibitors for industrial applications. Consequently, many researchers are focused on developing efficient, cost-effective materials to protect metals. In this study, an ecofriendly chitosan methionine derivative (M) was developed, synthesized, characterized, and tested for its anticorrosion properties. The ability of this compound as a corrosion inhibitor for carbon steel (CS) was confirmed through weight loss measurements (WL), potentiodynamic polarization (PDP), and electrochemical impedance spectroscopy (EIS) studies in a 1.0 M hydrochloric acid solution. The findings showed that the inhibitor, M, achieved a maximum inhibition efficiency of 99.8% at a concentration of 100 ppm by the PDP method. Additionally, the corrosion potential value, being less than 85 mV, supported classifying M as a mixed-type inhibitor with a cathodic tendency. The adsorption behavior of the inhibitor on the CS surface was consistent with Langmuir’s adsorption isotherm. EIS data also confirmed that increasing inhibitor concentration raised the charge transfer resistance (*R*_ct_), indicating improved protection. Surface examination using scanning electron microscopy (SEM) and energy-dispersive X-ray spectroscopy (EDX) revealed the formation of a protective layer of the M molecules on the CS surface. Moreover, theoretical studies, including analyses of the highest occupied molecular orbital (EHOMO), lowest unoccupied molecular orbital (ELUMO), dipole moment (µ), were thoroughly examined. Overall, both experimental and theoretical findings demonstrate that this derivative can effectively form a protective layer and mitigate corrosion.

## Introduction

Corrosion in the oilfield leads to various problems such as leaks in tanks, pipelines, and equipment^1–4^, leading to costly shutdowns that consume 20% of the annual maintenance budget. While acids are essential in industrial applications like pickling, and cleaning, they can damage steel equipment, particularly carbon steel^5–7^. Carbon steel is widely used for its corrosion resistance in engineering, oil production, and construction^8–10^. However, carbon steel’s susceptibility to corrosion remains a challenge, through it is manageable with proper treatment^11,12^.

Despite being a common issue, the corrosion of carbon steel is a treatable problem. One of the most significant additives for preventing carbon steel corrosion is inhibitors^13^. Organic inhibitors, particularly those containing nitrogen, oxygen, sulfur, and phosphorus, are widely used combat metal corrosion. This approach helps mitigate significant economic losses and prevent serious industry accidents caused by corrosion^14–19^. Previous studies have demonstrated that hetero-atoms, conjugate π bonds, and aromatic nuclei, in organic compounds are particularly effective in preventing corrosion^20–24^. These compounds reduce the corrosion rate by adsorbing onto the metal surface, where they block active sites by displacing water molecules^25–27^.

Unfortunately, many organic inhibitors are toxic, expensive, non-biodegradable, and harmful to the environment. With increasing environmental awareness and adverse effects of certain chemicals, recent research has focused on developing inexpensive, non-toxic and environmentally friendly, and biodegradable corrosion inhibitors. Naturally occurring polymers can readily meet these requirements and have demonstrated effectiveness in inhibiting metal corrosion in various aggressive environments^28^.

Polysaccharide-based polymers are regarded as promising natural inhibitors due to their active sites that can interact with metal ions. Chitosan, a notable polysaccharide, is particularly effective for use as an inhibitor on metallic surfaces. Various studies have reported that amino acids, natural polymers, medicinal drugs exhibit efficient inhibition of the corrosion process. Recently, a number of natural polymers such as pectin, starch, guar gum, gum Arabic have been investigated as corrosion inhibitors in different corrosive environments^3,29^ .

The subject of the present investigation, a chitosan derivative, is fully or partially deacetylated product of chitin, the second most abundant natural resource after cellulose. Chitosan is a natural biopolymer composed of ß-D-glucosamine and *N*-acetyl-ß-D-glucosamine units with a 1, 4-linkage. Due to its biocompatible, biodegradable and minimal toxicity, chitosan is Widely used in the pharmaceutical field as a drug delivery carrier and as a biomedical material^30^.

Moreover, chitosan exhibits various biological activities, including immunological, antibacterial, and wound healing properties, and has been proposed for tissue engineering applications. Beyond its medical applications, chitosan is also used in cosmetics, textile, paper, food and various other industrial sectors^31^. Analyzing the molecular structure of chitosan reveals the presence of amino and hydroxyl groups, which facilitate ionic interactions with metal surfaces. This characteristic enables chitosan to meet an important criterion for functioning as a corrosion inhibitor^32–34^.

However, chitosan is poor water solubility can limit its effectiveness as a metal corrosion inhibitor. To address this, derivatives of chitosan that have better water solubility can be used. These derivatives maintain the number of adsorption sites present in chitosan, potentially enhancing its efficiency as a corrosion inhibitor^35^. Additionally, chitosan’s ease of chemical functionalization enhances its adhesion to metallic surfaces. As a result, chitosan derivatives with specific functional groups and chitosan composites are preferred for corrosion protection over pure chitosan^28,36^.

Cheng et al.^37^ studied the use of carboxymethyl chitosan, a natural polymer, as an eco-friendly inhibitor for mild steel in HCl solution. The corrosion inhibition of mild steel in aerated 3% NaCl solution using chitosan-crotonaldehyde Schiff’s base investigated^38^. Acetyl thiourea chitosan has been reported as effective corrosion inhibitor for mild steel in 0.5 M H_2_SO_4_ solution^39^.

Metal corrosion occurs through electrochemical reactions at metal/solution interface, making electrochemical experiments and weight loss techniques ideal for studying corrosion. Electrochemical experiments provide authentic results in a shorter time frame. The adsorption film’s characteristic, which supports the inhibition mechanism, are explored in detail through adsorption and kinetic modeling weight losses method.

The novelty of our work lies in developing an environmentally friendly, cost-effective corrosion inhibitor with excellent protection efficiency, by synthesizing a soluble chitosan methionine derivative (M) suitable for use in acidic environments, thereby expanding its industrial application potential. This study evaluates the efficiency of the synthesized chitosan derivative (M) on carbon steel in hydrochloric acid using both electrochemical and weight loss measurements, providing comprehensive understanding of its performance across various methods. We also explore the relationship between inhibition efficiencies obtained experimentally and theoretical insights from quantum calculations, offering a deeper molecular-level analysis of its mechanism. This comprehensive approach enhances our understanding of this inhibitor’s potential for scalable industrial use.

## Experimental

.

### Material

All the chemicals utilized were of analytical reagent grade and were employed without additional purification. For immersion studies, commercially available carbon steel (CS) strips were sheared into dimensions of 5 cm × 2 cm × 2 mm, while for electrochemical studies, they were cut into 2 × 1 cm. The composition of the CS strips was determined to be as follows: Fe 99.3; C 0.218; Si 0.0198; Mn 0.188; P 0.0091; S 0.0246; Ni 0.0256; Al 0.0456; Cu 0.0376; Cr 0.0189; Mo 0.0089. prior to experimentation, the samples were abraded using successively finer grade emery papers, washed with absolute ethanol, rinsed with doubly distilled water, dried, weighed, and stored in desiccators for subsequent use. Analar grade hydrochloric acid (Merck) was employed to prepare the corrosive medium, with 1 M solutions being prepared using double distilled water.

###  Preparation of chitosan methionine

Chitosan (1.0 g) was added to 30 ml deionized water and allowed to swell overnight. Subsequently, alpha amino gamma-methyl thiol butyric acid (methionine) (0.5 g) was slowly added while stirring. At room temperature the mixture was stirred for 24 h, and half of the solvent was then removed under the reduced pressure. Anhydrous ethanol was gradually added to the solution until no fibrous precipitate formed. Centrifuging at 3500 rpm for 5 min and the precipitate residue was washed twice with ethanol. After vacuum drying, off-white powders were obtained^40^. The structure of chitosan M was generated using the software ChemDraw Ultra Version7.0.

### Characterization

#### Fourier transform infrared (FT-IR)

The structure of the prepared chitosan derivative M was measured by Fourier transform infrared spectrometer—Perkin–Elmer 1750, in range of 400 –4000 cm^− 1^. The IR image in this study was generated using the software Origin Pro Version 8.5.

#### Nuclear magnetic resonance (NMR)

Bruker Advance II 400 NMR spectrometer was used to determine the ^1^HNMR spectra of chitosan derivative. The NMR image in this study was generated using the software MestReNova Version 14.2.1-27684.

#### Thermal analysis (TGA and DTA)

Thermal analysis of M was performed by thermo-gravimetric analyzer model TGA TA-51 at 20 °C/min in a nitrogen atmosphere. The TGA image in this study was generated using the software Sigma plot version 10.

### Corrosion analysis

The experiments were conducted in 1 M HCl solution with and without different concentrations (25–100 ppm) of the prepared Chitosan derivative M. The images in this part were generated using the software Sigma plot version 10.

#### Weight loss measurements (WL)

The weighed samples were immersed in 100 ml of 1 M HCl solution, both with and without different concentrations of M (ranging from 25 to100 ppm) at temperature ranging from 298 to 328 K. After maintaining the specified immersion time and temperature, the specimens were removed, dried in moisture-free desiccators and re-weighed using a sensitive analytical balance Model FA 2104 A (cap.: 210 g, d.: 0.1 mg). To ensure the reproducibility, the experiments were conducted in triplicate. The difference in weight before and after immersion was considered as the weight loss, which was utilized to calculate the corrosion rate.

The corrosion rates were calculated according to Eq. (1)^5^ :

1 $$\:{C}_{R}=\frac{\Delta W \times 534}{Atd}$$

where *C*_*R*_ is the corrosion rate (mpy), *∆W* (g), is the difference in the specimen weight before and after immersion, A is the surface area of CS specimens (cm^2^), t is the exposure time (*h*) and d is density of CS in g/cm^3^.

The degree of surface coverage (*θ*) was calculated using Eq. (2)^41^

2 $$\:\theta=\frac{{W}_{o}-{W}_{i}}{{W}_{0}}$$

where, *W*_i_ and *W*_o_ are the weight losses of CS specimens in inhibited and uninhibited solutions, respectively. The inhibition efficiency *IE*% was calculated according to Eq. (3):^42^.

3 $$\:IE \%= \theta \times 100$$

#### Electrochemical corrosion tests

Electrochemical measurements were conducted in a conventional three-electrode glass cell using Potentiostat/ galvanostat 302 supported by Nova software Version 1.11. A carbon steel CS specimen with an exposed area of 1 cm^2^ served as working electrode, while silver/silver chloride electrode was used as reference electrode, and a platinum electrode as the counter electrode. The electrochemical tests were performed at room temperature (298 k) after reaching a steady-state potential.

##### Potentiodynamic polarization (PDP)

PDP studies were performed by scanning the potential in the range ± 250 mV versus open circuit potential (OPC) at a sweep rate of 1 mVs^− 1^. The anodic and the cathodic branches of the PDP curves were extrapolated until they intersected to determine the corrosion potential (*E*_*corr*_), the corrosion current density (i_corr_), and polarization resistance (*R*_*p*_ ). the corrosion inhibition efficiency (*IE %*) was calculated using an Eq. (4)^43^.

4 $$\:IE\%=\frac{{I}_{o}-{I}_{i}}{{I}_{0}} \times 100$$

where, I_o_, I_i_ are the current densities in absence and presence of M.

##### Electrochemical impedance spectroscopy (EIS)

EIS measurements were conducted at the open circuit potential by applying a frequency range of 100 kHz to 0.01 Hz with a10 mV amplitude. The corrosion inhibition efficiency was evaluated using the polarization resistance (*R*_P_)^44^ .

5 $$IE\% = \frac{{{R_1} - {R_0}}}{{{R_1}}} \times 100$$

### Surface studies

The influence of the corrosive attack on the CS surface was investigated using a scanning electron microscope (SEM), Quanta 250 FEG, FEI, Hillsboro, Oregon) at high vacuum chamber conditions (4.8 × 10 − 6 Torr pressure), 20 kV acceleration voltage. CS samples were prepared to the specified surface finish and then immersed in 1 M HCl solution period of 6 h, both with and without 100 ppm of M inhibitor.

### Density functional theory (DFT)

DFT is the basis for all computations involving quantum chemicals^45^ Gaussian 09 09^46^, the Lee–Yang–Parr (B3LYP) correlation functional^47^ on the 3-21G basis set^48^, and the B3LYP hybrid exchange correlation (local, nonlocal) were all employed in the computations. Chem Bio Draw Ultra 12.1 was used to generate a 2D synthetic structure^49^. Quantum chemical properties, such as the highest occupied molecular orbital (HOMO) and the lowest unoccupied molecular orbital (LUMO), have been identified. The dipole moment (µ), energy gap (ΔE), electronegativity (χ), chemical hardness (η), global softness (σ), electrophilicity index (ω), number of electrons transported (ΔN), and back-donation energy (Eback-donation) were all taken into consideration when determining the results of DFT calculations. The image was generated using the software Gauss View 5.0.9 program.

## Results and discussion

**Fig. 1:**
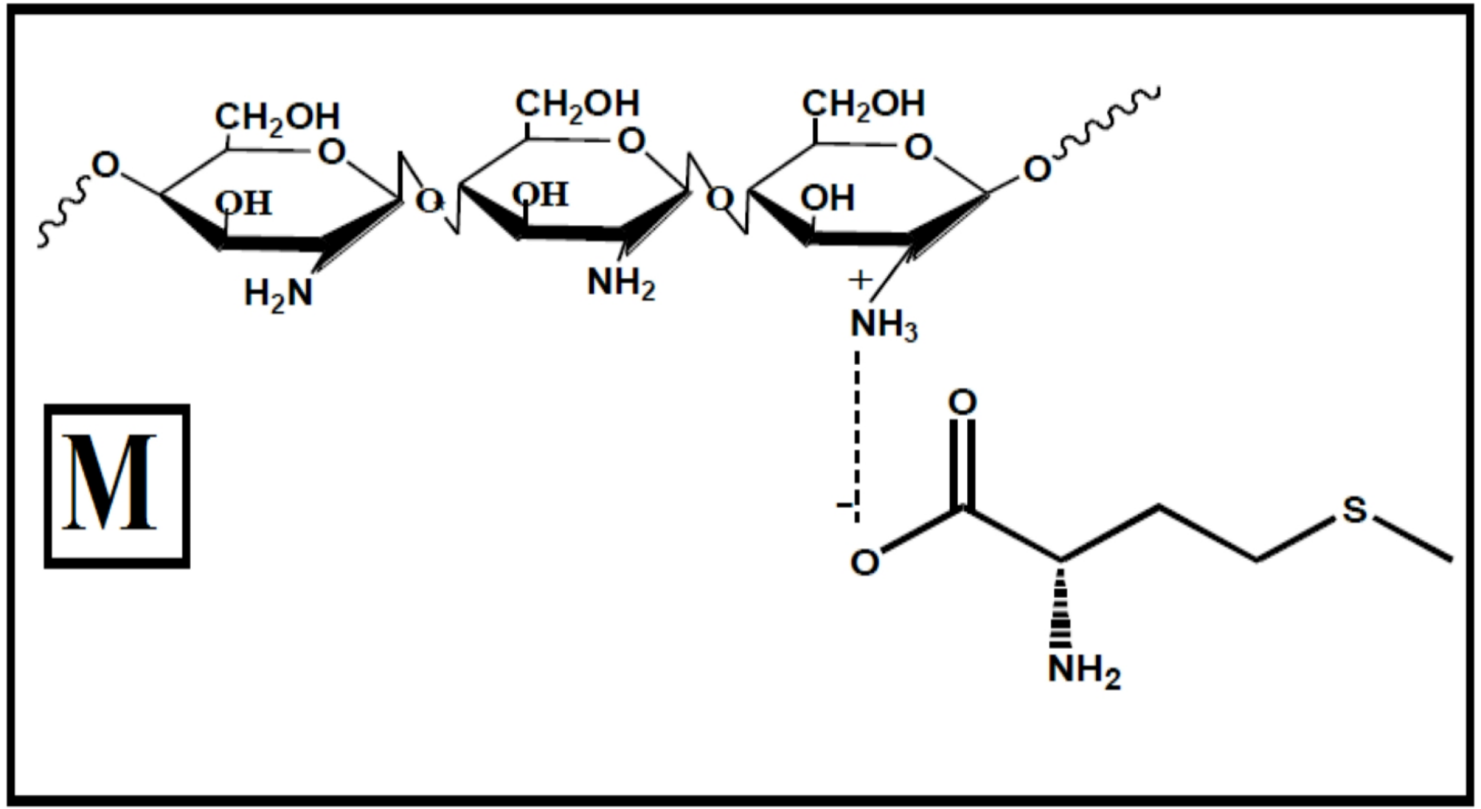
Structure of chitosan methionine (M) conjugate complex.

The active sites on chitosan include the amine and hydroxyl groups. As a result, chitosan derivatives have been synthesized and applied to inhibit metals and alloys in in aggressive environments. One of the modifications involves the formation of chitosan derivative (M), as depicted in Fig. 1, through the reaction of an alpha amino gamma methyl thiol butyric acid (methionine) with the free amino group of chitosan, which improve chitosan’s functionality and expand its application scope.

### Structural analysis Chitosan methionine (M) conjugate complex

The characterization of the synthesized inhibitor was performed by using FTIR spectroscopies and H-NMR as well as thermal analyses.

#### FT- IR spectra

**Fig. 2:**
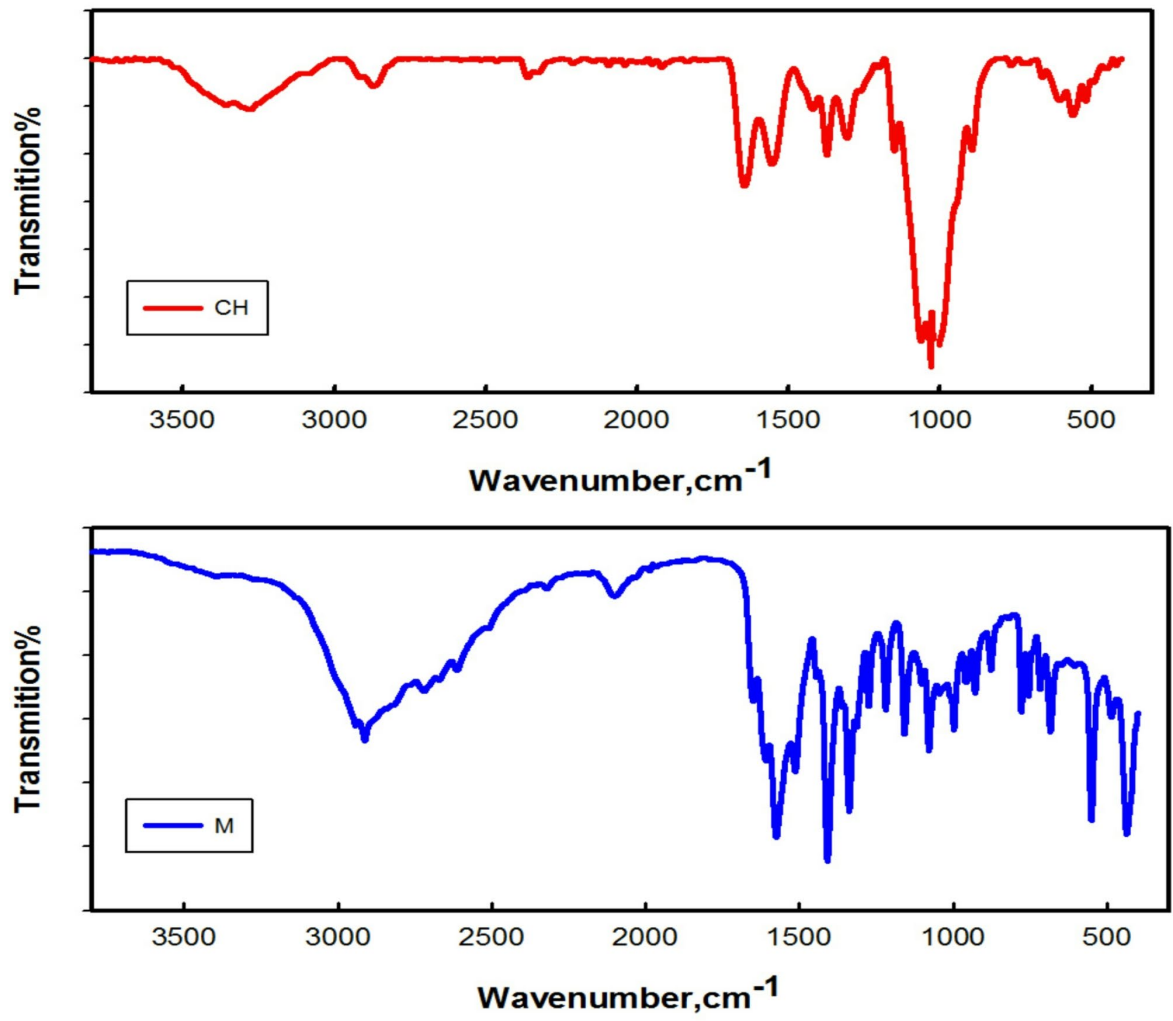
The FTIR spectra of chitosan (CH) and chitosan methionine (M) conjugate complex.

The functional group of chitosan M was examined using Fourier transform infrared spectroscopy (FT-IR). In Fig. 2 illustrate the IR spectral analysis of chitosan (CH) and its derivative M. A broad band at approximately 3330 cm^− 1^ corresponds to stretching vibrations of –NH and −OH groups a characteristic feature of chitosan’s polysaccharide structure. The typical absorption peaks of amide I (C=O) and amide II (–NH_2_) bands are observed at around 1655 and 1536 cm^− 1^, respectively^50^. Bands within the range of 1150–898 cm^− 1^ signify the polysaccharide backbone, including the glycoside bonds, C–O–C and C–O stretching.

In the spectra of chitosan amino acid salts (M), the wider and weaker band at about 3375 cm^− 1^ for (M) compared with chitosan, this indicating the formation of –NH_3_^+^ in the molecule. The absorption peaks of methylene group (–CH_2_ – stretching vibration) in methionine are observed at 2930 cm^− 1^, strong peaks at approximately 1569 cm^− 1^ corresponds to stretching vibrations of (C=O), and strong peak at 1079 cm^− 1^ was assigned to C–S in methionine^39^. In summary, the IR spectra suggest that chitosan methionine formed successfully by protonation reaction.

#### ^1^H-NMR

**Fig. 3:**
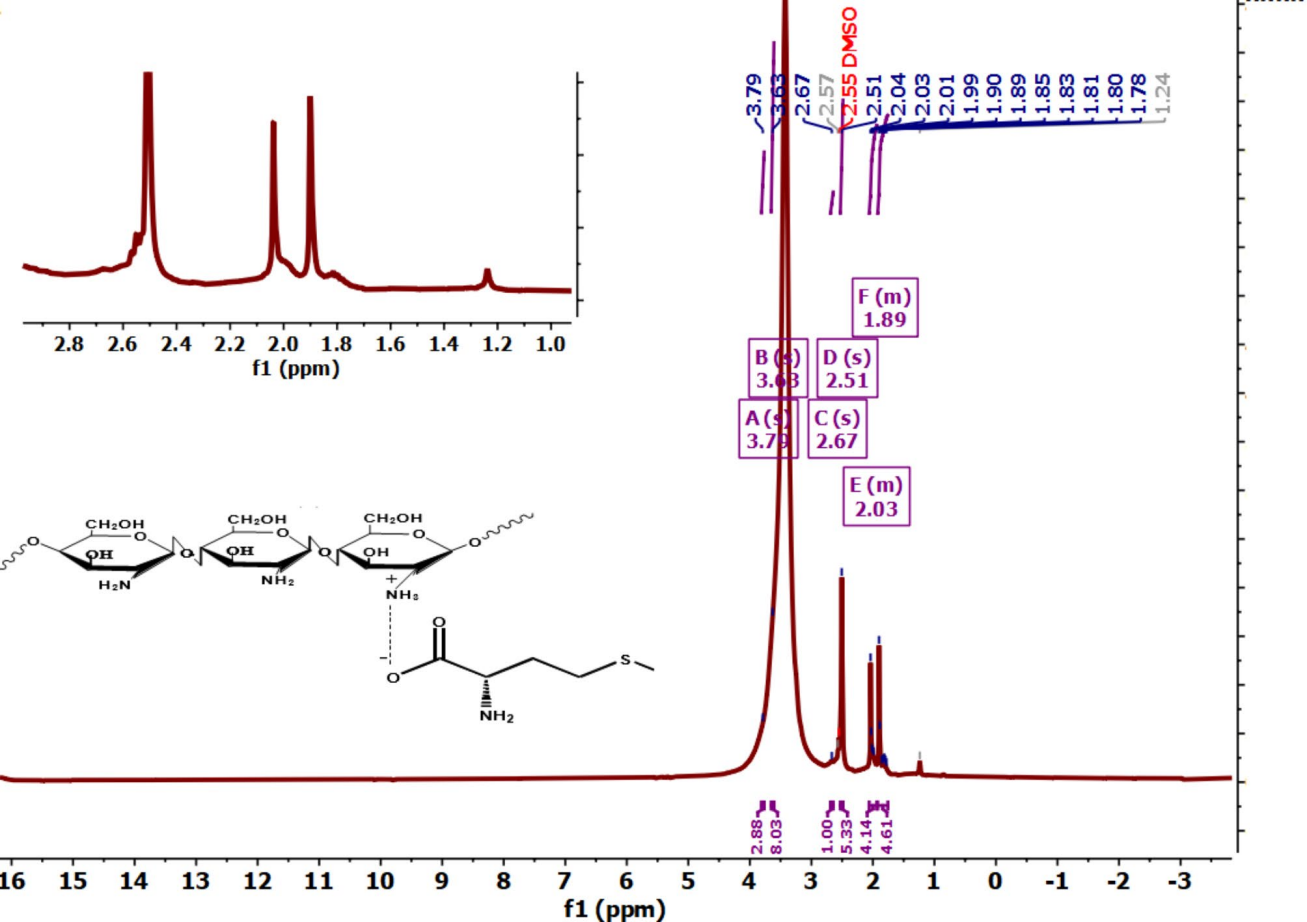
^1^H NMR spectra of chitosan and chitosan methionine conjugate complex in set.

The ^1^H-NMR spectrum of chitosan and chitosan methionine (M) is depicted in Fig. 3. in which the concentrated chemical shift from 3.1 ppm to 4.1 ppm was due to the chemical environment of the protons in Chitosan chains^51^. The 1HNMR spectrum of methionine (M) conjugate complex is illustrated in the inset picture of Fig. 3, in which the additional peaks from 1.89 ppm to 2.03 ppm clearly indicated the conjugation of methionine groups to the backbone of Chitosan. Furthermore, protons from the DMSO solvent appear at a chemical shift of 2.5 ppm.

#### Thermal analysis

**Fig. 4:**
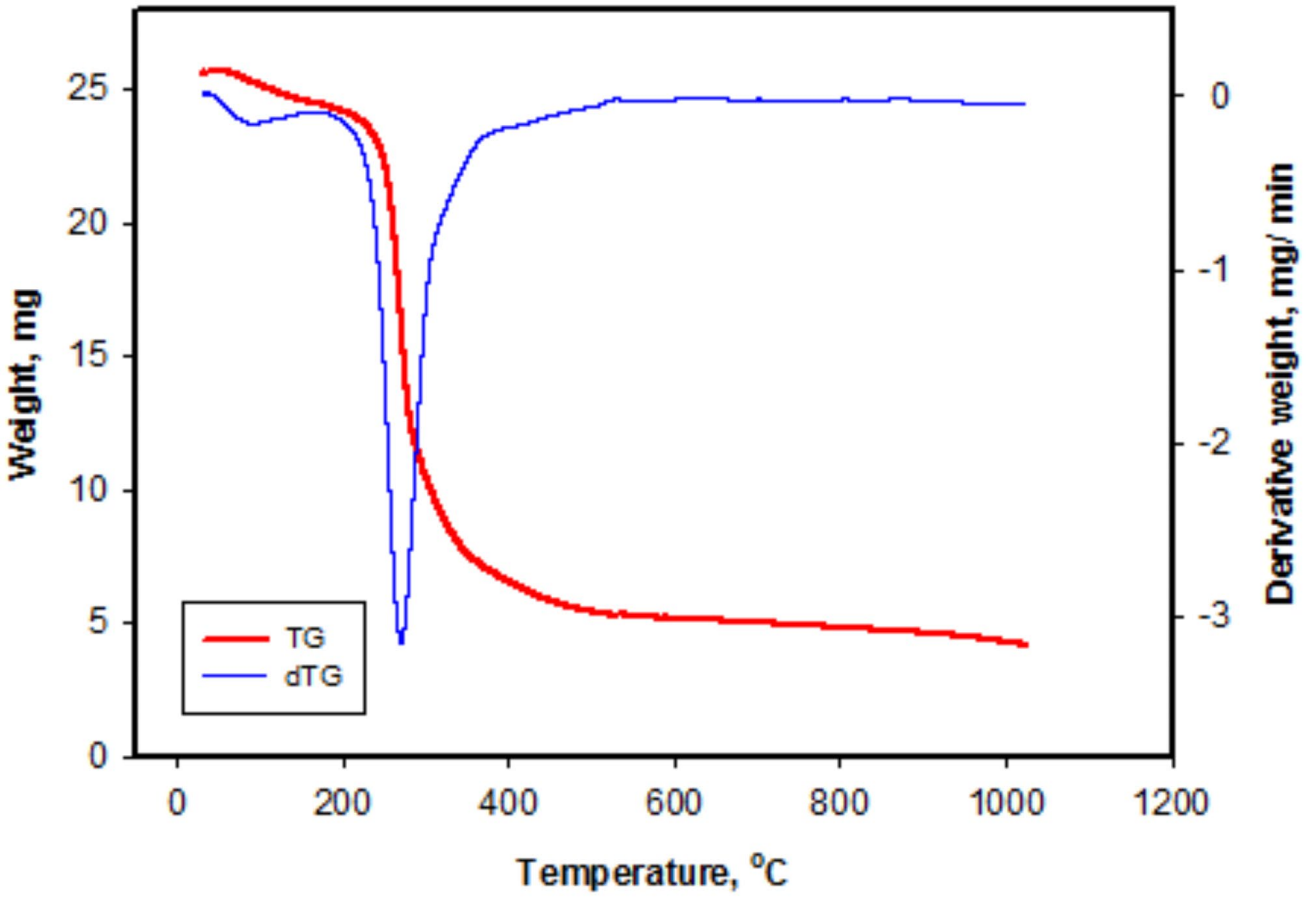
TGA and DTA of chitosan methionine (M) conjugate complex.

The thermal analysis shown in Fig. 4 includes thermogravimetric analysis (TGA) and differential thermal analysis (DTA) of the chitosan methionine conjugate complex. The TG curve illustrates the thermal decomposition of the chitosan methionine compound. It is evident that the TG curve exhibits a smooth profile with three distinct degradation patterns. The first degradation, occurring from 99.4 °C to about 281 °C, is likely due to dehydration or the elimination of remaining solvent. The second one, starting at 282 °C, corresponds to the breakdown of the main polymeric chains. The third weight loss is observed between 543 °C and 976 °C.

However, it is noteworthy that the weight loss of chitosan is higher than the prepared complex^52^.This suggests that the incorporation of methionine reduced the weight loss and improved the thermal stability of chitosan methionine.

### Weight loss measurements

#### Influence of inhibitor dosage

**Fig. 5:**
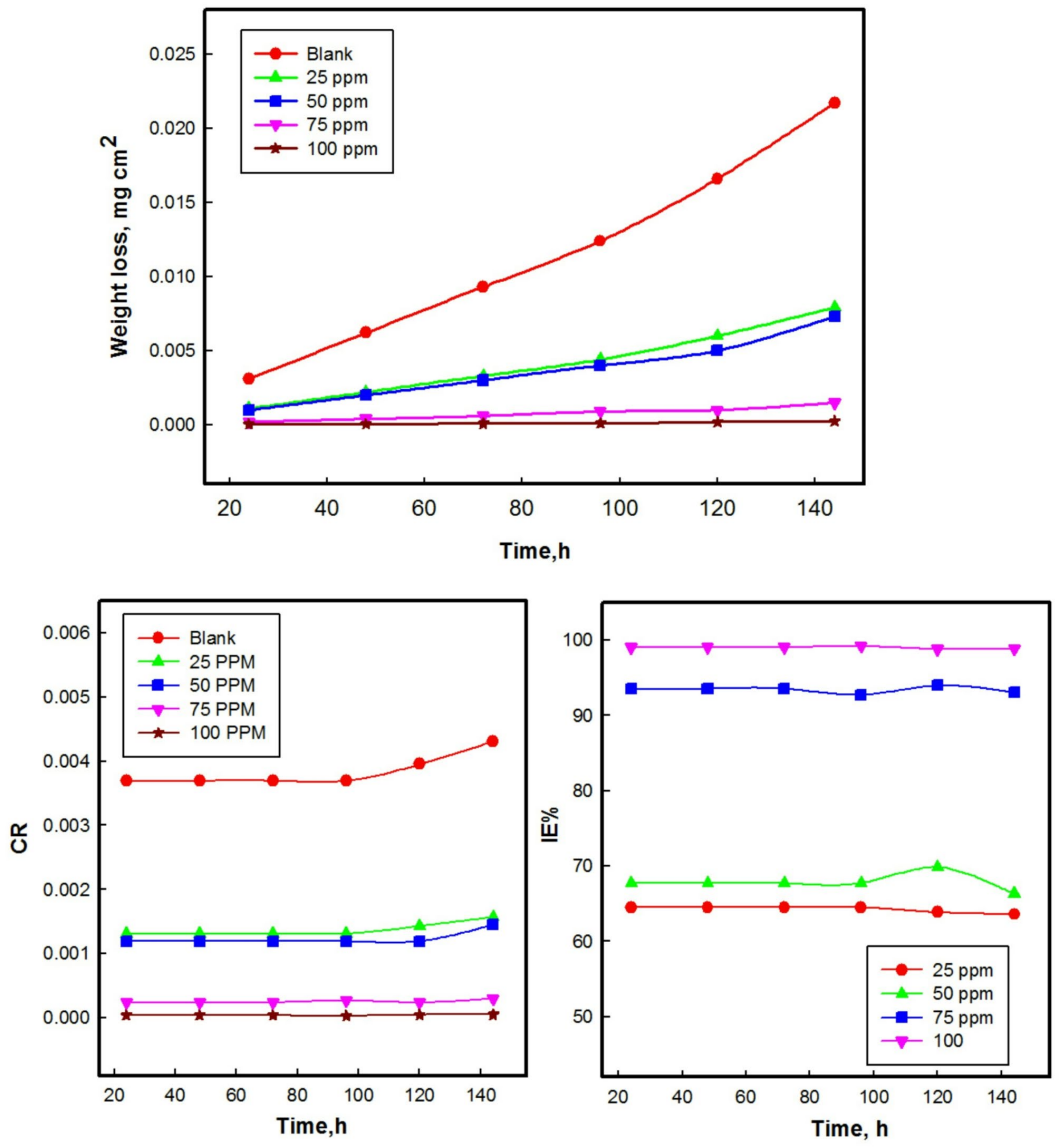
Variation of weight loss, corrosion rate (CR) and inhibition efficiency (IE %) with time of CS in 1 M HCl with and without different concentrations of M at 298 K.

The weight loss experiment was conducted in 1 M HCl solution both in the absence (blank) and presence of various concentrations of M ranging from 25 to 100 ppm. The weight loss data obtained from these experiments was utilized to calculate the corrosion rate and the inhibition efficiency using Eqs. (1–3). The variation with time is illustrated in Fig. 5.

The weight loss of CS in the presence of inhibitor exhibits a linear variation with time, consistently lower than that of the blank solution. Additionally, there is an observed increase in weight loss with an increase in immersion time. This trend is accompanied by a decrease in efficiency, may be attributed to the desorption of protective layer^53^.

The increase in the inhibitive performance of chitosan derivative M with the increase in concentration implies that higher concentrations of the inhibitor lead to more effective protection against corrosion, likely due to increased adsorption of the M molecules on the CS surface forming a more robust layer.

These findings suggest that M effectively inhibits metal corrosion across all studied concentrations. The results indicate that CS experiences significant corrosion uninhibited 1 M HCl solution. However, increasing the concentration of M considerably reduce the dissolution and corrosion rate, while also enhancing inhibition efficiency. This suggests a strong interaction between the inhibitors and metal surface^54^, rendering M effective as an inhibitor for CS in 1 M HCl. The observed reduction in corrosion rate and weight loss can be attributed to the adsorption of the inhibitor compounds onto the surface of the CS, thereby blocking sites and facilitating the formation of a protective film on the surface.

The notable efficiency of compounds is attributed to the presence of adsorption sites in the organic compounds, which facilitate favorable adsorption^55^. At the optimum inhibitor concentration of 100 ppm, a remarkable 98.84% inhibition efficiency is achieved. This high level of inhibition efficiency underscores the effectiveness of the inhibitor in mitigating corrosion.

The results existing in Table 1, indicates that regardless of immersion time, the inhibition efficiency increases as the concentration of the inhibitor increases. This trend suggests that higher concentrations of the inhibitor lead to more effective inhibition of corrosion, consistently enhancing the protection afforded to the metal surface.

**Table 1:** Corrosion rate (CR), coverage surface (*θ*), CR, and IE% at various M concentrations for CS in 1 M HCl solution obtained by weight loss method at 298 K.

Concentration (ppm)	(CR) (mg cm^− 2^ h^− 1^)	(θ)	(IE %)
Blank	0.0086	–	–
25	0.0031	0.636	63.59
50	0.0029	0.664	66.35
75	0.0006	0.931	93.08
100	0.0001	0.988	98.84

#### Influence of temperature

**Fig. 6:**
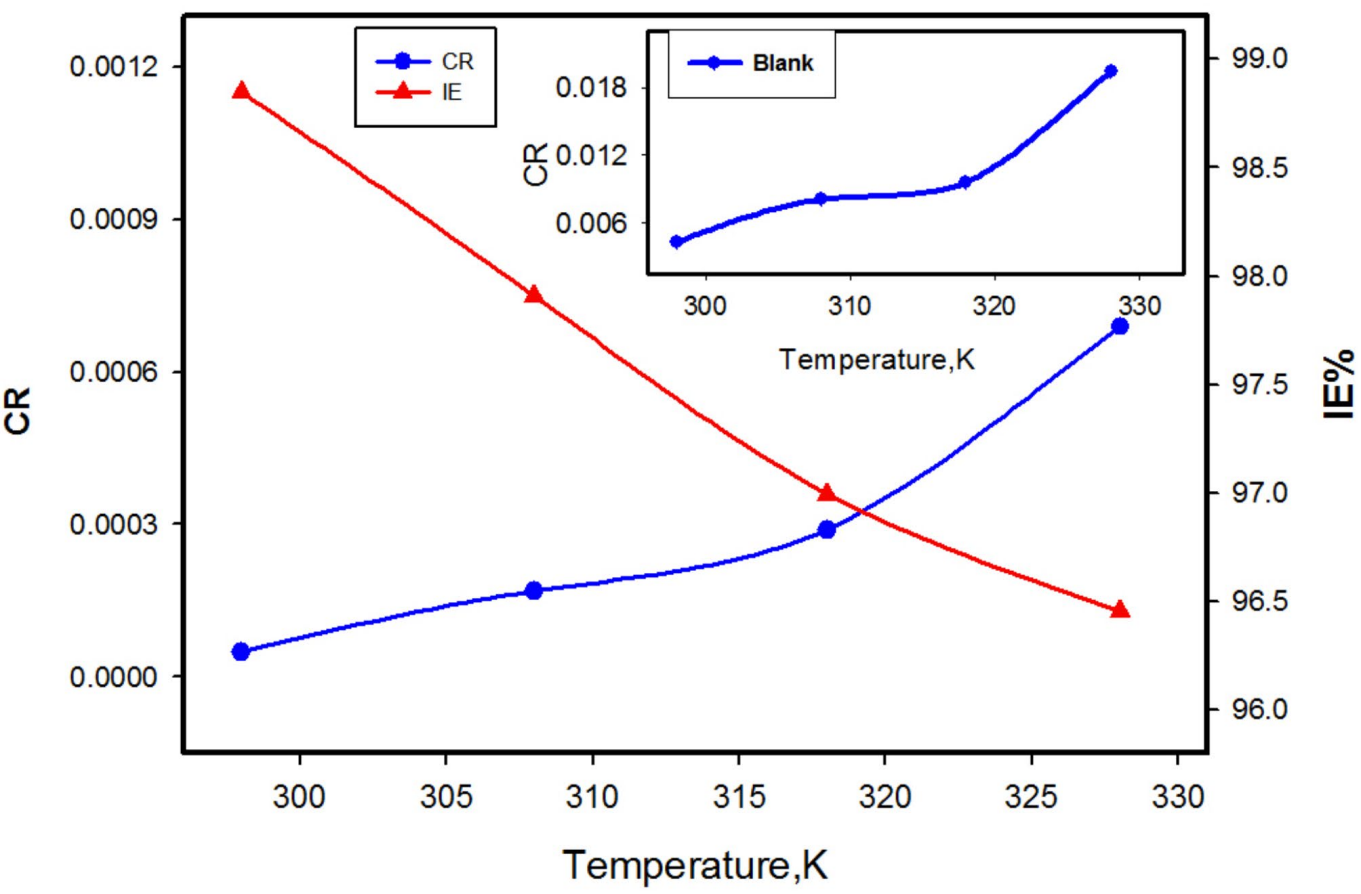
CR and IE% of CS in 1 M HCl with 100 ppm of M at temperature rang 298–328 K, CR without M inset curve.

Inhibition efficiency was measured by weight loss in the temperature range of 298–328 K for various inhibitor concentrations. The results depicted in Fig. 6 demonstrate that an increase in the temperature enhances the corrosion rate, consequently decreasing the efficiency. The reduction can be attributed to the heightened effect of temperature on the dissolution process of CS and partial desorption of adsorbed M molecules from the surface. This suggests that inhibitor adsorption occurs primarily through physisorption^56–58^. This phenomenon can also be explained based on structural orientation^59^. Alternatively, the polymeric molecules degraded into smaller segments, which then adhere to the active sites of the CS surface, leading to better inhibition efficiency^60^.

#### Adsorption isotherms and thermodynamic examination

##### Thermodynamic adsorption parameters

To obtain a thorough understanding of how inhibitor molecules (M) reduce CS corrosion, researchers designed multiple adsorption isotherms. These isotherms offer valuable insights into interactions between inhibitor molecules and the active sites of the metal surface^29^. The surface coverage θ is determined using Eq. (2), which derives from the weight loss data observed during the experiments.

**Fig. 7:**
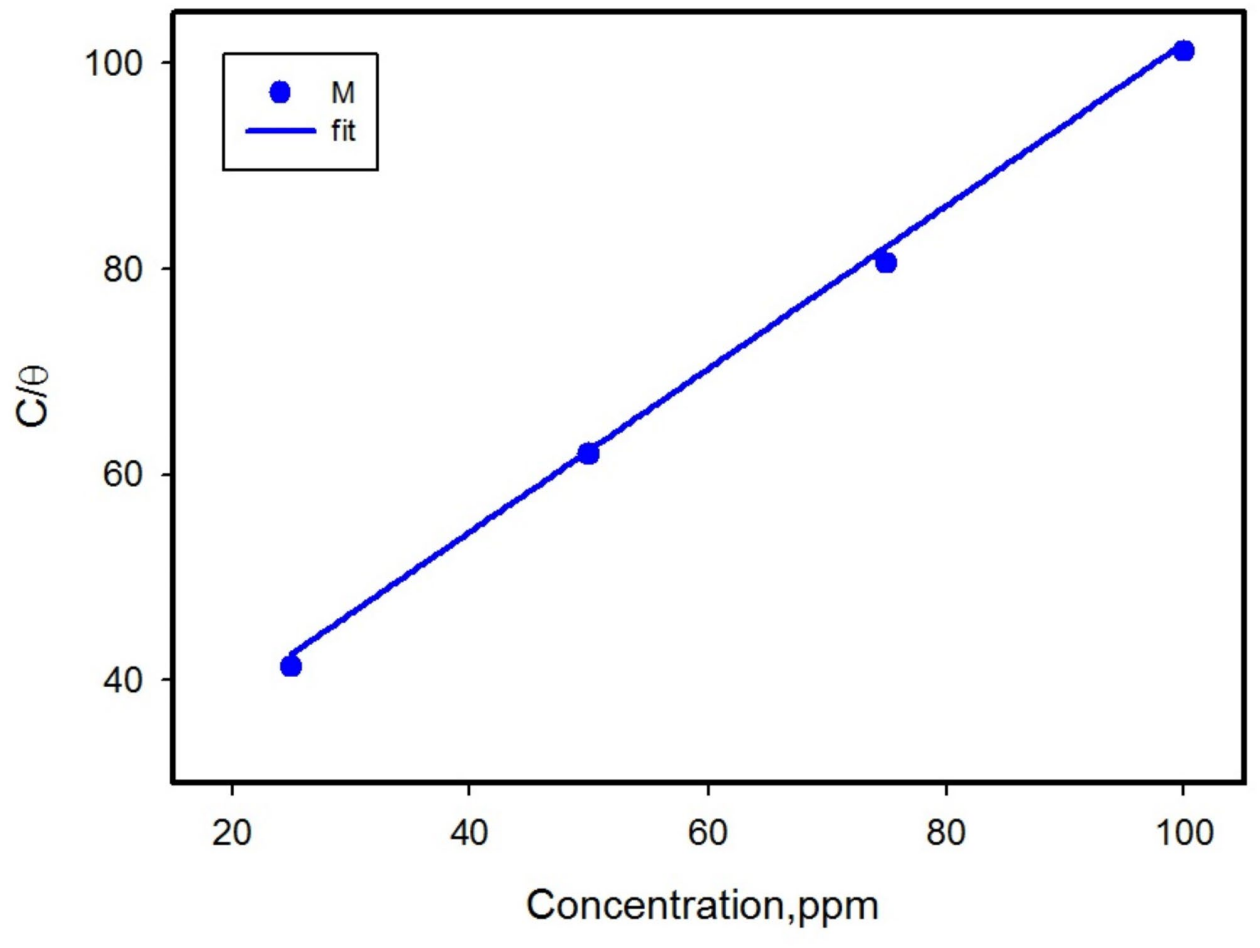
Langmuir adsorption isotherm of different M concentrations on CS in 1 M HCl.

**Fig. 8:**
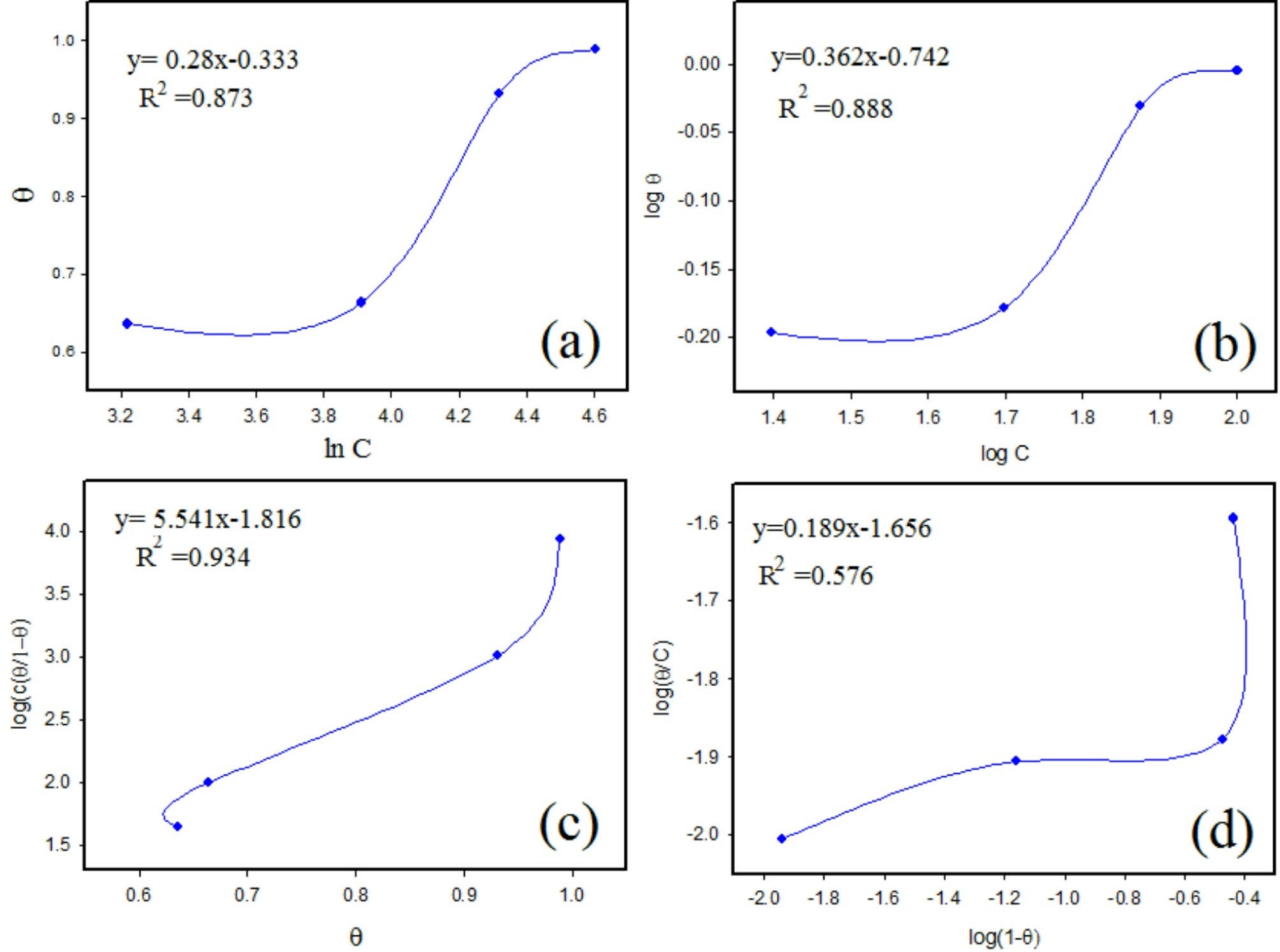
Adsorption isotherms of different M concentrations on CS in 1 M HCl (**a**) Temkin, (**b**) Frendlish (**c**) Frumkin (**d**) Fluri-Hugins.

In thermodynamic studies of adsorption isotherms, the goal is to understand how inhibitor molecules interact with active centers on metal surfaces to inhibit corrosion. This process involves fitting experimental data of surface coverage (θ) into various mathematical models of adsorption. These models include Langmuir, Temkin, Freundlich, Frumkin, and Flory-Huggins isotherms, and each offering different insight into the adsorption behavior of the inhibitor molecules. Figures 7 and 8 display the different isotherm models used. For more details, the corresponding parameters adsorption equilibrium constant (*K*_ads_) and regression coefficient (R^2^) for M were calculated and listed in Table 2. The increase in *K*_ads_ values indicates the power of the adsorption of M inhibitor molecules on the metal surface^57^.

The best-fit model is typically identified by the highest *R*^2^ value obtained from the linear plots. The Langmuir isotherm emerges as the best suited, with the highest *R*^2^ values as shown in Fig. 7; Table 2 Therefore, it is suitable for evaluating the adsorption equilibrium constant, *K*_*ads*_^61^.

**Table 2:** Different adsorption isotherms parameters for CS.

Isotherm	Equation^62^	K_ads_ (M^− 1^)	*R* ^2^
Langmuir	*C*/*θ* = C + (1/*K*) (6)	0.0379	0.935
Temkin	log *θ* = log*K*_ads_ + n logC (7)	− 0.2892	0.821
Freundlish	*θ* = lnC + *K*_ads_ (8)	0.1987	0.828
Frumkin	Log (c (*θ/1- θ*)) = *2α θ* + 2.303 log *K*_ads_ (9)	0.0458	0.916
Flory–Huggins	log *θ/*C = b log(1*- θ*) + log *K*_ads_ (10)	34.127	0.576

Furthermore, the determination of the significant variable, standard free energy of adsorption (Δ*G*^*o*^_ads_), is related to the arrangement of organic inhibitors on the metal surface in 1 M HCl medium. This is derived from Langmuir isotherm data according to Eq. (11):

11 $$\Delta {G^{\text{o}}}_{{\text{ads}}} = - RT{\text{ln}}\left( {{C_{\text{w}}}{K_{{\text{ads}}}}} \right)$$

where, *R* is the universal gas constant, and T is the temperature in K, and *C*_w_ is the water concentration equal 1 × 10^6^ is the concentration of water molecules denoted in mg L^− 1^^62^. The negative of ∆*G*_*ads*_ value indicate the spontaneous adsorption process. The magnitude of ∆*G*_ads_ equal to- 9.002 (kJ mol^− 1^) can be attributed to physisorption of M through electrostatic interaction between charged molecules and charged metal surface^63^. The enthalpy Δ*H*^o^_ads_ and the entropy Δ*S*^o^_ads_ of adsorption are calculated using the Gibbs–Helmholtz model, according to Eqs. (12) and (13):

12 $$\Delta {G^{\text{o}}}_{{\text{ads}}}T = \Delta {H^{\text{o}}}_{{\text{ads}}}T + {K_{{\text{ads}}}}$$

13 $$\Delta {G^{\text{o}}}_{{\text{ads}}} = \Delta {H^{\text{o}}}_{{\text{ads}}} - T\Delta {S^{\text{o}}}_{{\text{ads}}}$$

Δ*H*^o^_ads_ equals − 20.29 (kJ mol^− 1^),with the negative values reflecting the exothermic behavior of inhibitor on the CS surface^64^. Δ*S*_ads_ equals − 0.0378 (JK^− 1^ mol^− 1^), and the negative value indicates the decrease in the disorderly adsorption of inhibitor molecules on the metal surface^65^.

### Kinetic and thermodynamic corrosion parameters

**Fig. 9:**
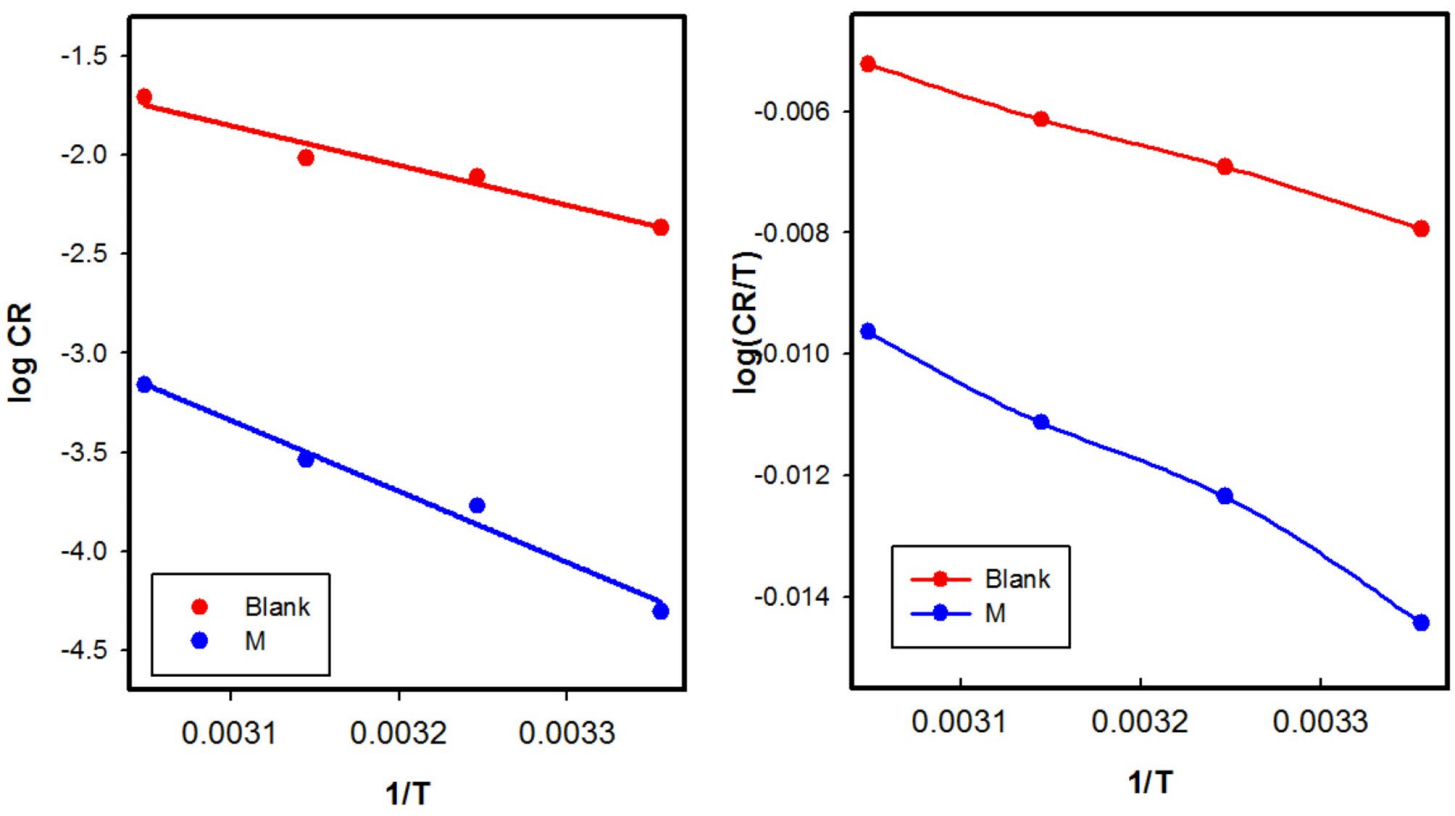
Arrhenius plot (**a**) and Transition state plot (**b**) for CS in absence and presence of 100 ppm of M.

The effect of temperature on the inhibited-acid metal is highly complex, as numerous changes occur on the metal surface, including rapid etching, desorption, and decomposition of the inhibitor^57^.The thermodynamic parameters for the dissolution of CS were calculated based on changes in the values of corrosion rate. Figure 9 shows the relationship between logarithms of the corrosion rate (CR) and the reciprocal of the absolute temperature (1/*T*) for CS corrosion in 1 M HCl in the absence and presence of 100 ppm of M chitosan. The activation energies (*E*_a_^*^) were calculated using Eq. (14):

14 $$\:log{C}_{R}=A-\left(\frac{{E}_{a}^{*}}{2.303\:RT}\right)$$

where A is the extrapolation factor, R is the universal gas constant.

Figure 9a gives straight lines with a correlation coefficient of 0.99, and the slope *E*_a_*/2.303*R*, from which E_a_ was calculated. From Table 3 both inhibited and uninhibited solution, the *E*_a_^*^ value is greater than 20 kJ/mol which illustrating that the entire process is controlled by surface reaction. For the uninhibited solution, the value of *E*_a_^*^ was 38.06 kJ/mol and the addition of 100 ppm of M raises *E*_a_^*^ to 68.61 kJ/ mol signifying physical adsorption of M on the CS surface. With increasing the desorption, more surface area of the metal is exposed to acid attack, leading to an increase in corrosion rate at elevated temperatures, resulting in decreased protection efficiency^29^. Figure 9b shows the plot of log *C*_R_/*T* against 1/*T* in the absence and presence of 100 ppm.

To calculate the enthalpy ∆H* and entropy ∆S* of activation for the corrosion process the Eyring transition state (Eq. 15) was used:

15 $$\log \frac{{{C_R}}}{T} = \log \left( {\frac{R}{{Nh}}} \right) + \left( {\frac{{\Delta {S^*}}}{{2.303R}}} \right) - \left( {\frac{{\Delta {H^*}}}{{2.303RT}}} \right)$$

where *h* is the Planck’s constant, 6.23 × 10^− 34^ J⋅s, *N* is Avogadro’s number 6.022 × 10^23^ mol^− 1^. Slope of (∆H*/2.303*R*) and intercept of [log (*R*/*Nh*)+∆S*/2.303 *R*)] from which the values of Standard enthalpy of activation (∆H*) and standard entropy of activation (∆S*) were calculated and given in Table 3.

The more positive values of ∆H* can be associated with endothermic adsorption, indicating that the slower dissolution of CS in inhibited solution compared to uninhibited one. This suggests that the decrease in corrosion rate is primarily controlled by the kinetic parameters of activation. The less negative values of ∆S* for inhibited solutions, in comparison to the blank solution, indicate a decrease in disordering of the activated complex^44^. In other words, one could state that the Fermi energy level of the CS surface increased because of the inhibitor molecules that were adsorbed onto it^66^.

**Table 3:** Activation and thermodynamic parameters of CS in absence and presence of 100 Ppm of M.

Concentration (ppm)	$\:\varvec{A}$	Ea* (KJ mol^− 1^)	∆H* (KJ mol^− 1^)	∆S* (KJ mol^− 1^ K^− 1^)	*R* ^2^
Blank	4.31	38.06	1.61	− 197.19	0.975
100	7.77	68.61	0.29	− 196.87	0.986

### Chemical kinetics of corrosion inhibition

**Fig. 10:**
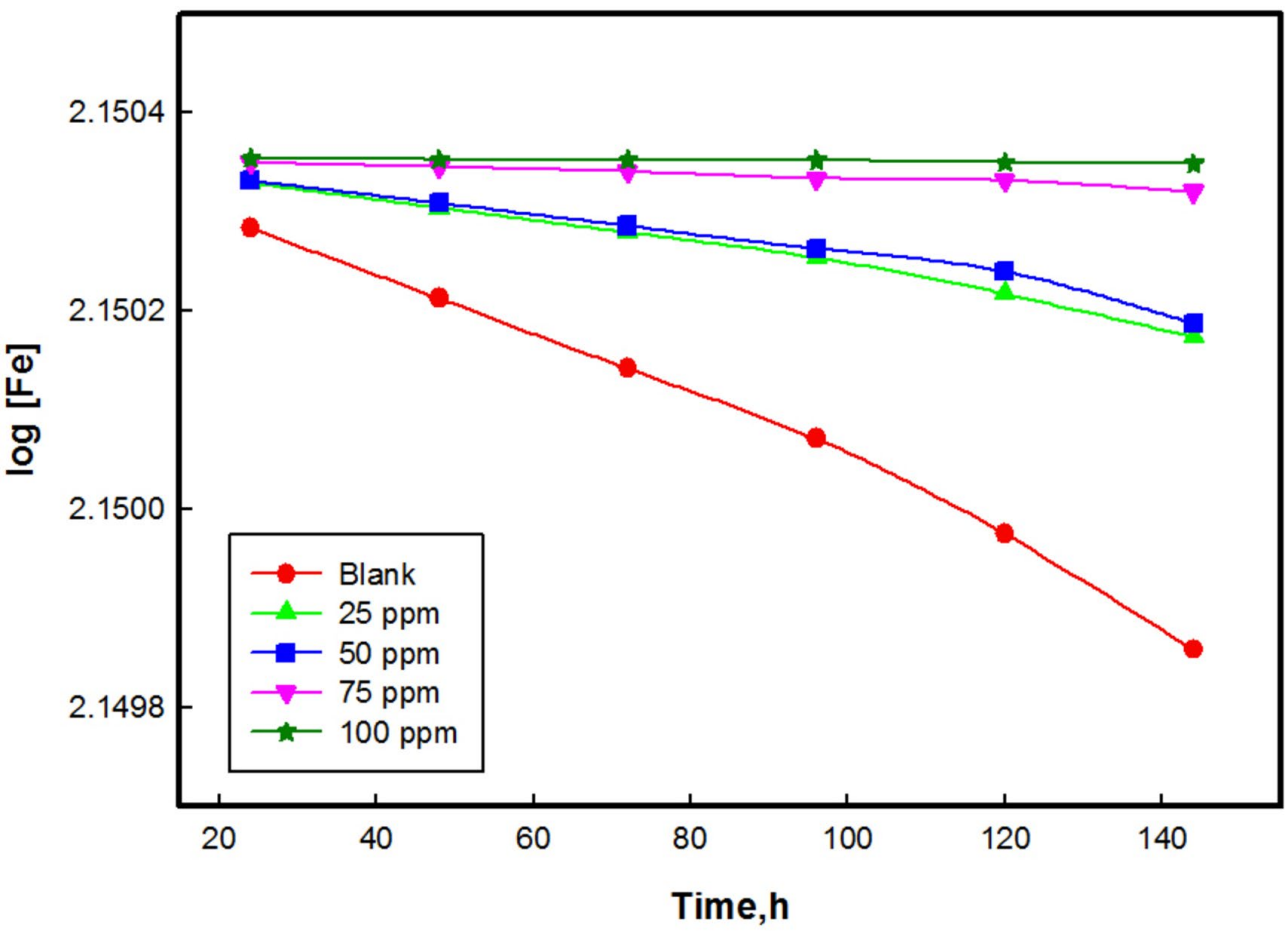
Chemical kinetic plot for the corrosion of CS with and without different concentrations of M.

The kinetics can be described by relating the molar concentration of the CS to the immersion time. Assuming a (mol/L) is the initial concentration of the CS and *x* (mol/L) is the final concentration of CS that has decomposed into corrosion products after time t. Consequently, the concentration of CS remaining at time *t* is (*a–x*) mol/L. The experimental results show that plotting of either log (*a − x*) or log [CS] on Y-axis against time on abscissa gives straight lines with regression coefficient (*R*^2^) values close to unity. This confirms that the reaction follows first-order kinetics (Fig. 10). Equation (16) represents the first-order rate equation:

16 $$\:-\text{log}\left[\text{c}\text{o}\text{r}\text{r}\text{o}\text{d}\text{e}\text{n}\text{t}\right]=\frac{{K}_{1}t}{2.303}$$

where k_1_is the first order rate constant and t is the time in hours. Also, the half-life (t_0.5_) of a first order reaction is related to the rate constant according to Eq. (17):

17 $$\:{t}_{0.5}=\frac{0.693}{{K}_{1}}$$

The Table 4 records the values of the rate constants (*K*_*1*_) and half-lives (*t*_*0.5*_) obtained from the kinetic plots. The findings indicate that the rate constant (*k*_*1*_) of CS in the presence of M is lower than that of the blank solution. Additionally, the half-lives of CS in the presence of M is longer compared to the half-life in the blank solution. These results suggest that the solutions containing compound M effectively increase the half-life of CS in 1 M HCl, indicating inhibition of corrosion.

From the weight loss measurements over time, it is evident that the corrosion rates and half-life for metal dissolution are inhibited to a significant extent. A good inhibitor is characterized by its ability to prolong the time taken for metals to convert into corrosion products. Therefore, M is considered a good corrosion inhibitor based on these results^67^ .

**Table 4:** Chemical kinetic parameters for CS corrosion in 1 M HCl in absence and presence of different concentrations of M.

Conc. (ppm)	K (min^− 1^)	$\:{\varvec{t}}_{0.5}$ (min)	*R* ^2^
Blank	6.91 × 10^− 6^	1.00 × 10^5^	0.988
25	2.30 × 10^− 6^	3.01 × 10^5^	0.985
50	4.61 × 10^− 6^	1.50 × 10^5^	0.968
75	4.61 × 10^− 7^	1.50 × 10^6^	0.966
100	9.21 × 10^− 8^	7.52 × 10^6^	0.922

### Electrochemical measurements

#### Open circuit potential (OCP)

The OCP signifies the potential of the working electrode concerning that of the reference electrode when no applied potential or current is present. It’s essential to measure this before conducting EIS and PDP measurements.

**Fig. 11:**
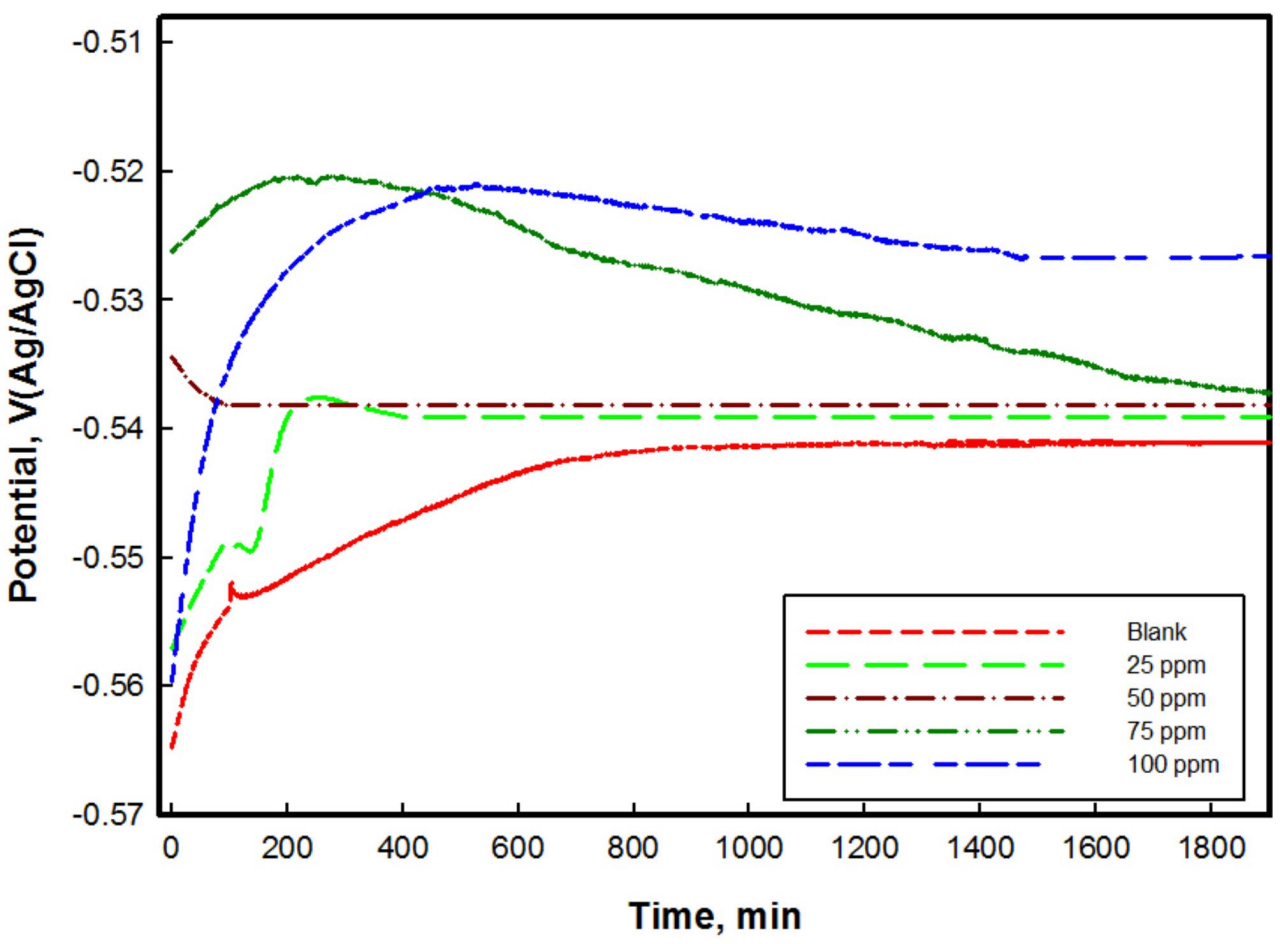
Open circuit potential for CS in presence of various concentrations of chitosan M derivative at 298 K.

Figure 11 illustrates the changes in the OCP of CS over time in 1 M HCl with and without different concentrations of M for 1 h. A noticeable alteration in the behavior of the CS was observed. The curves indicate that the 15 min were sufficient to reach a steady-state potential. In the presence of M, the OCP of CS shifted towards more positive values compared to its absence. The steady values for the CS substrate is − 0.541 V shifted to − 0.520 V more noble direction with addition of M. The less negative potential indicates a lower tendency for corrosion. The most efficient concentration for protection is 100 ppm due to the highest positive potential shift. The adsorbed chitosan M effectively isolates CS surface from the surrounding environment.

#### Potentiodynamic polarization method

**Fig. 12:**
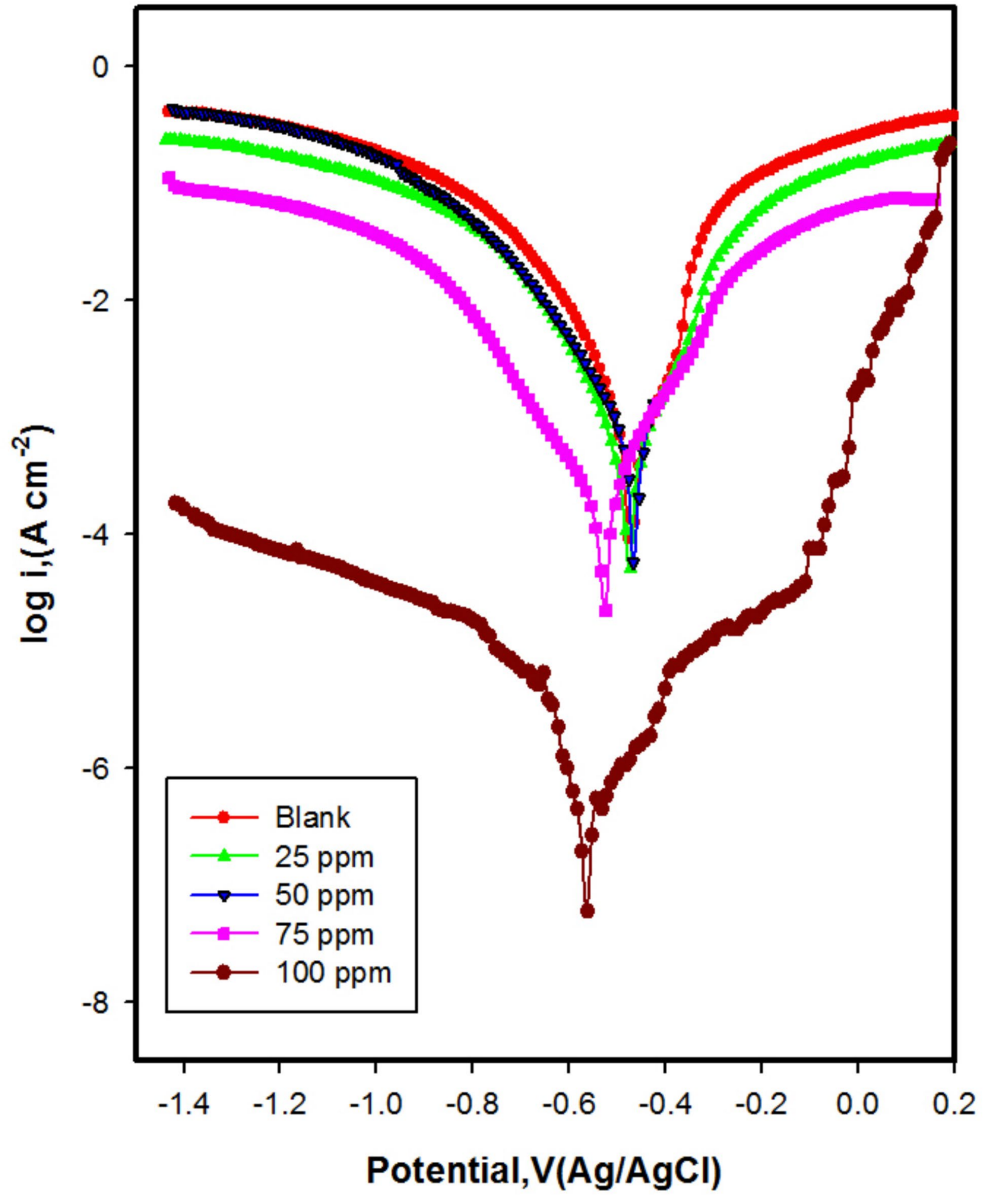
Polarization curves for CS in absence and presence of various concentrations of M at 298 K.

To validate the data obtained from weight loss, potentiodynamic experiments were conducted. Figure 12 displays the polarization curves for CS in 1 M HCl with and without varying concentrations of chitosan M. Table 5 lists the electrochemical parameter such as corrosion potential (*E*_corr_), anodic and cathodic Tafel slopes (*β*_a ,_*β*_c_), corrosion current density (*I*_corr_), polarization resistance *R*_pol_, corrosion rate (CR), and inhibition efficiency (IE %). The decrease in corrosion current density (*I*_corr_) with increasing concentration of chitosan M from 25 to 100 ppm, the ( *I*_corr_) decreased from 4.0 × 10^− 4^ to 4.75 × 10^− 7^ A cm^− 2^, leading to decrease the corrosion rate, suggests the blocking of active sites of CS by the adsorption of M molecules^68,69^.

The corrosion potential without inhibitor equal − 0.472 V shifted to more cathodic value equal − 0.561 V in presence of optimum concentration 100 ppm of chitosan M. The corrosion potential value being less than 85 mV further supports the classification of chitosan M as mixed type inhibitor with a cathodic tendency. It could suggest that the inhibitor is blocking the cathodic reaction (hydrogen reduction), making the cathodic process slower and thereby protecting the steel from corrosion^70,71^.

The anodic (βa) and cathodic (βc) Tafel slopes are changed in the presence of M inhibitor, as shown in Table 5. This behavior can be explained on the basis that a protective layer formed on the metal surface^72^. Higher β_a_​ or β_c_ in presence of inhibitor than in absence indicates slower reaction rates due to inhibition. Furthermore, based on the calculated Tafel slopes, increasing the chitosan M concentration (25 to 100 ppm) raised the polarization resistance from 47.25 Ω cm^2^ in blank solution to 62,599 Ω cm^2^ in the presence of 100 ppm chitosan M, confirming the anticorrosion behavior.

Utilizing Eq. (4), the inhibition efficiency increased with increasing chitosan M reached 99.8% at 100 ppm chitosan M.

**Table 5:** The polarization parameter values for CS in 1 M HCl with and without different concentration of M.

Conc. (ppm)	E_corr_ (V(Ag/AgCl))	β_a_ (V dec ^1^)	β_c_ (Vdec^1^)	I_corr_ (Acm^− 2^)	*R*_*P*_ (Ohm. cm^2^)	CR	IE (%)
Blank	− 0.472	0.077	0.112	4.17 × 10^− 4^	47.5	4.84	–
25	− 0.479	0.124	0.115	4.0 × 10^− 4^	64.70	4.67	35.00
50	− 0.458	0.061	0.108	2.65 × 10^− 4^	49.74	3.09	36.10
75	− 0.525	0.110	0.162	1.54×x10^− 4^	185.30	1.79	63.01
100	− 0.561	0.212	0.201	4.75 × 10^− 7^	62,599	0.0055	99.80

####  Electrochemical impedance spectroscopy (EIS)

**Fig. 13:**
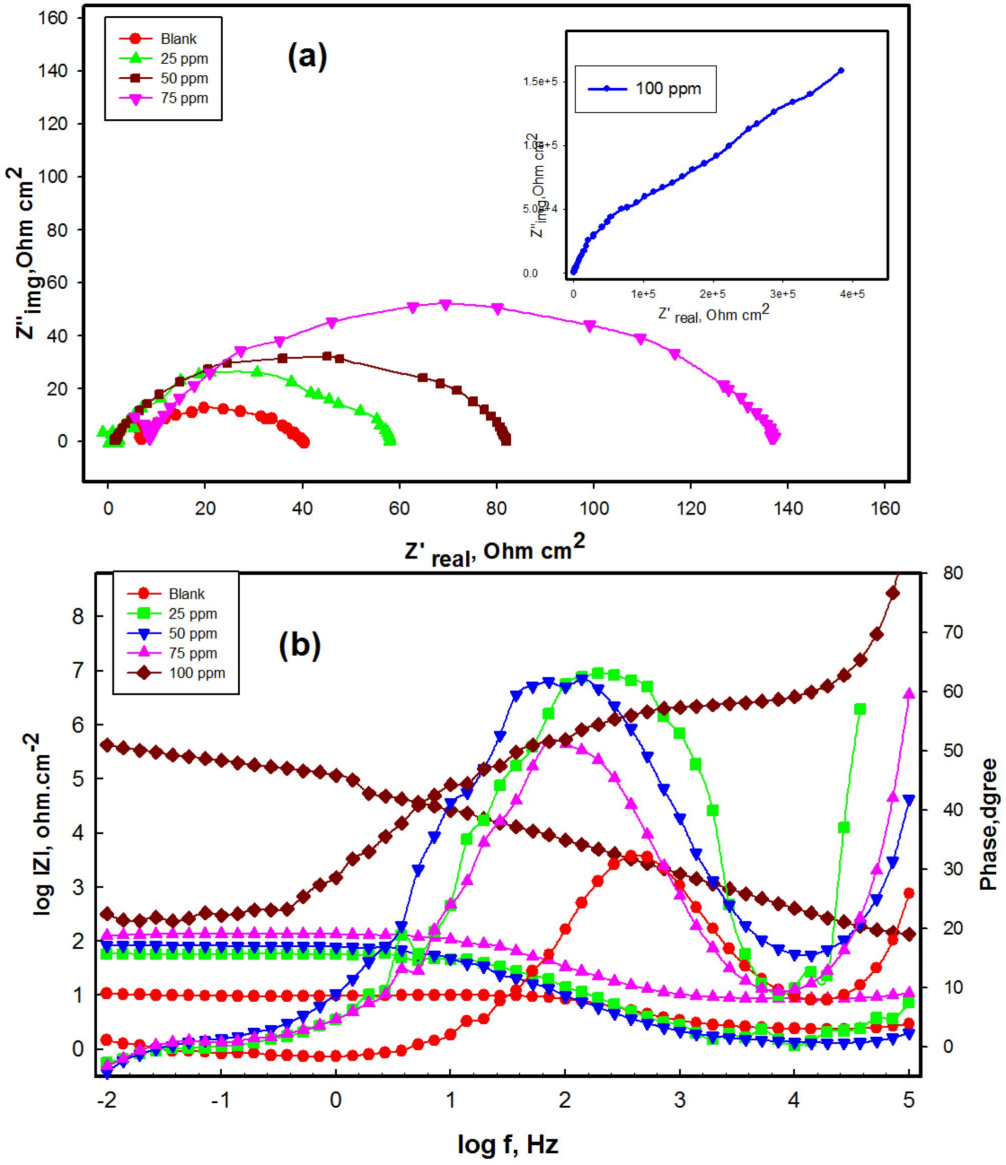
EIS plots for CS in presence of various concentration of M at 298 K (**a**) Nyquist plots, (**b**) bode and phase plots.

Non destruxtive EIS results are deemed more consistent because the measurements were taken at the steady state potential, making it a non-destructive^73^. Figure 13a illustrates the Nyquist plot for CS in 1 M HCl in the absence and presence of M. The Nyquist plots reveal a single capacitive loop, and the semicircle diameter increases with a rise of M concentration, recommend the formation of a protective film, enhancement the corrosion resistance^29,74^. The experimental EIS data, presented as Bode and phase plots show the impedance magnitude ∣Z∣ as a function of frequency and phase angle as function of frequency in Fig. 13b. At low frequencies, the Bode plots reveal a resistive region characterized by a horizontal line and a phase angle near zero. log|Z| becomes constant its value increases with increase concentration from 2.4 Ohm cm^2^ to 5.6 Ohm cm^2^, with phase angle values approaching zero. Higher impedance values at low frequencies typically indicate stronger barrier to charge transfer, meaning less corrosive attack, more effective inhibitor. The phase angle increase in presence of M compared to blank. High-frequency region related to solution resistance; suggest surface roughness or inhibitor adhesion.

**Fig. 14:**
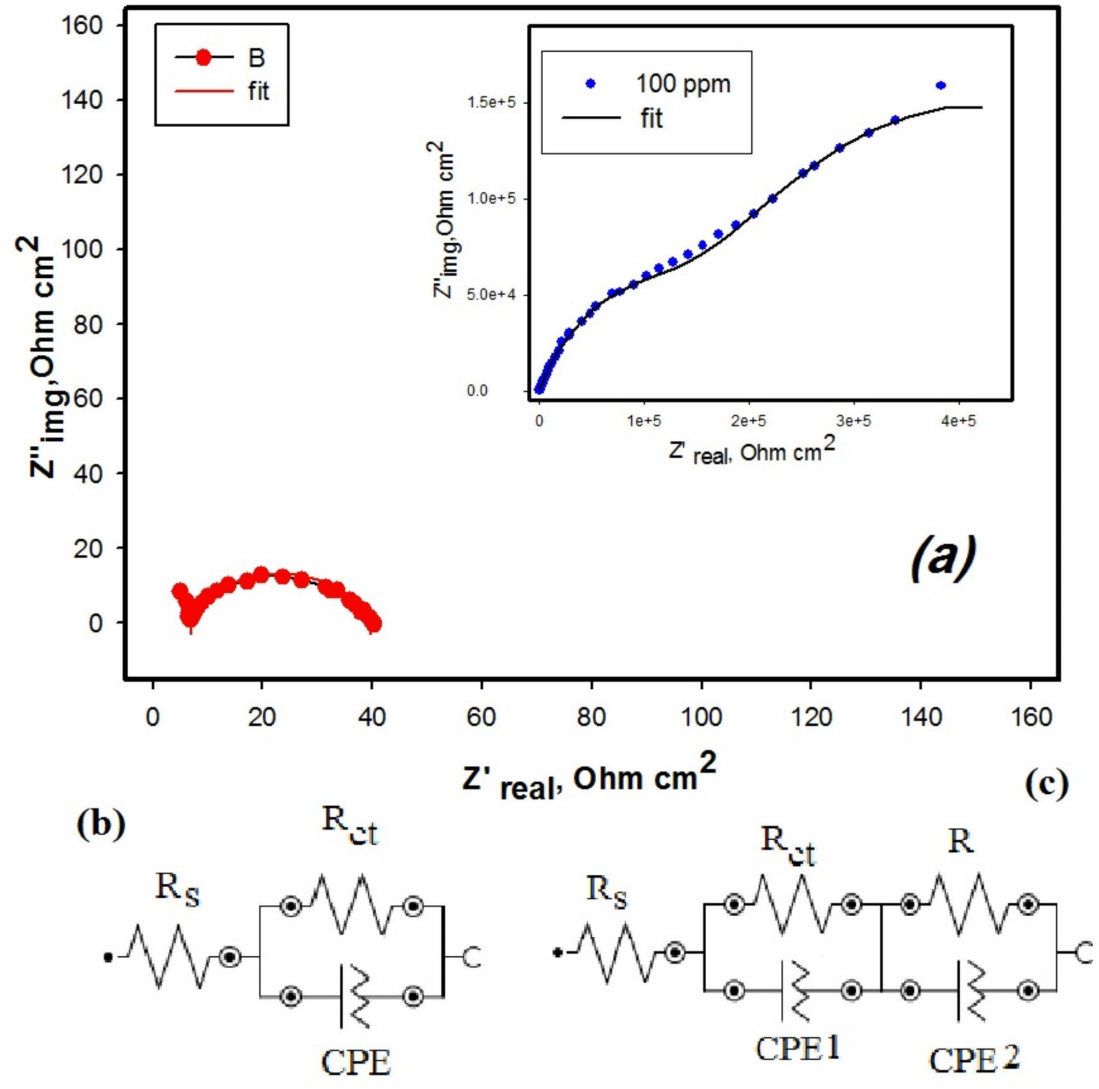
(**a**) fitting curve for blank and optimum concentration 100 ppm and the equivalent circuit (**b**) for blank and (**c**) for 100 ppm chitosan M.

Figure 14a show the fitting curves for blank solution and 100 ppm chitosan M. EIS parameters were determined using the equivalent circuit as seen in Fig. 14b,c. *R*_s_ is the ohmic resistance between the reference and the working electrode; *R*_*ct*_ denotes the charge transfer resistance. The electrical double-layer capacitance (*C*_dl_)^41^, is expressed in terms of a constant phase element (CPE), due to the inhomogeneity, roughness, and adsorption. The charge transfer resistance R_ct_ values for the inhibited solution are observed to be higher compared to the uninhibited one and increase with an raise in inhibitor concentration^57^.

The decrease in CPE values occurs due to an increase in the thickness of the electrical double layer^58^, attributed to the adsorption of the M molecules via donor-acceptor interaction between the lone pair of nitrogen atoms .Creating protective layer that reduce the contact of corrosive agents to the CS surface by forming a strong hydrophobic barrier due to the long hydrocarbon (–CH_2_) chain. The efficiency depends on the surface coverage and concentration of adsorbed inhibitor molecule on the metal surface. The fitting quality of the suggested circuit has been verified by the chi-square (χ^2^) values listed in Table 6. A smaller χ^2^ value indicates a better fit of the recommended equivalent circuit, and fit results match well with the experimental data.

Utilizing Eq. (5) the corresponding efficiencies were measured and reported in Table 6^44^.

**Table 6:** EIS parameters of CS in 1 M HCl in the absence and presence of a various concentration of M.

Conc., ppm	*R*_s_ (Ωcm^2^)	*R*_CT_ (Ωcm^2^)	Y_o_ (Fcm^2^)	*n*	*R* (kΩ cm^2^)	Yo (Fcm^2^)	*n*	IE%	X^2^
Blank	2.35	29.7	2.57 × 10^− 4^	0.873	–	–	–	–	0.41
25	1.75	55.9	2.19 × 10^− 4^	0.814	–	–	–	46.8	1.41
50	1.26	82.3	2.18 × 10^− 4^	0.819	–	–	–	63.9	1.14
75	7.90	138.4	1.51 × 10^− 4^	0.822	–	–	–	78.5	1.72
100	1.47	143,570	1.33 × 10^− 6^	0.614	565.2	9.41 × 10^− 6^	0.601	99.8	0.43

### Surface analysis

#### Scanning electron microscopy (SEM)

**Fig. 15:**
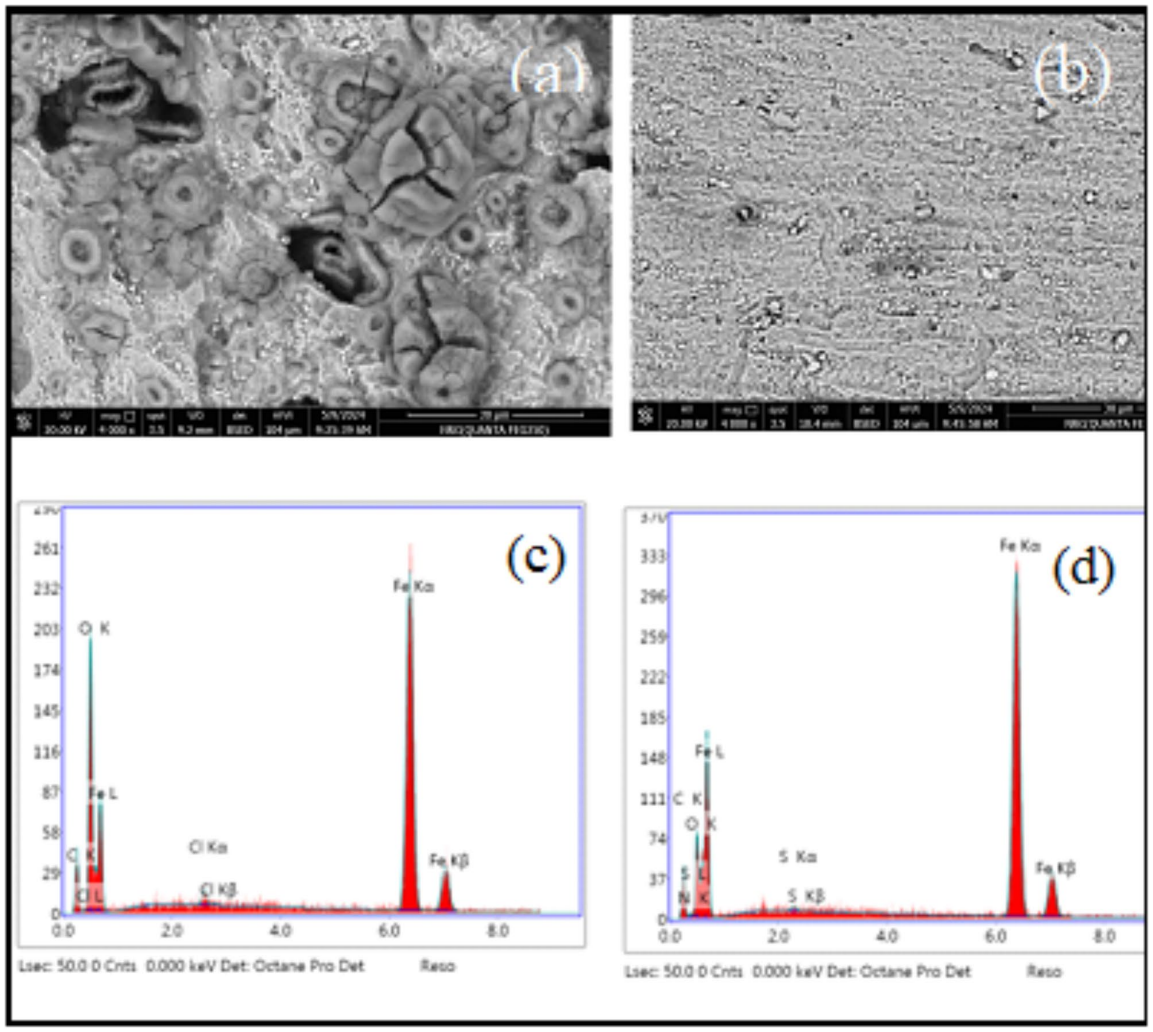
SEM images and EDX spectra of CS (**a**,**c**) in 1MHCl (**b**,**d**) in 200 ppm M.

Figure 15a,b displays the SEM of CS in 1 M HCl both in absence and presence of 100 ppm of M. A comparison of the corroded rough surfaces of CS in 1 M HCl alone with CS immersed in the inhibitor solution reveals smoother surfaces in the latter, as observed in a detailed analysis of the SEM pictures. This finding suggests that the presence of the inhibitors significantly reduces *CR*, likely due to the inhibitor adhering to the surface and forming a protective layer^3^.

#### Energy‑dispersive X‑ray spectrometer (EDX)

Figure 15c,d depicts the spectrum of the CS surface both in absence and presence of the M inhibitor. The spectrum of CS in absence of M contains (Fig. 15c) mainly shows peaks corresponding to Fe and O with minor quantities of Cl. This suggests that the corrosion of iron occurred, leading to the formation of iron chlorides and/or iron oxides. The intensity of oxygen molecules is observed to be higher than that of carbon atom, indicating the prevalence of iron oxides on the surface.

In contrast, the protective layer analyzed by EDX in the presence of M inhibitor revealed the presence of carbon, oxygen, sulfur and iron (Fig. 15d). The detection of carbon, oxygen and iron on the surface indicates unquestionably the presence of M molecules on the CS surface. The oxygen peak observed in the EDX spectra can be attributed to the oxygen atoms present in the inhibitor, which likely coordinate with iron. Additionally, the presence of carbon in the EDX profile confirms the adsorption of M molecules on CS steel surface. This adsorption prevents iron corrosion by blocking weak points on the surface and hindering the formation of ferric and ferrous chloride compounds.

### Inhibition mechanism

When carbon steel specimen is immersed in an aqueous solution, the anodic reaction Fe → Fe^2+^ + 2e ^−^ and the cathodic reaction is 2 H^+^ + 2e^−^ → 2H_2_. Organic inhibitors mitigate corrosion by adsorbing onto the metal/solution interface, with their efficiency determined by molecular structure, metal surface charge, and charge distribution.

The chemical structure of the chitosan M contains N, S, O heteroatoms along with chitosan repeating units with –NH_2_ and –OH groups. It could be assumed that, NH_2_ group can be protonated in acid media and the cationic form of M may adsorb on the cathodic sites of CS surface. Protonated amine groups interact electrostatically with the negatively charged metal surface, facilitated by chloride ions, while heteroatoms (N, O, S) with lone pairs adsorb on anodic sites, and OH groups interact with cathodic sites. Adsorption can occur directly via electrostatic attraction or through bridges formed by pre-adsorbed acid anions. Experimental findings show that compound M significantly improves corrosion inhibition in 1 M HCl due to its nitrogen, oxygen, and sulfur units, which enable strong adsorption, especially through the C–S bond. Sulfur, being a stronger electron donor than nitrogen, adsorbs more effectively in acidic environments, creating a robust protective layer. Chitosan, in its protonated and neutral forms, facilitates adsorption through electrostatic interactions, while chloride ions enhance the inhibitor’s adsorption efficiency. The superior performance of M stems from its ability to form a compact, stable adsorbed layer on the metal surface, with sulfur and nitrogen atoms playing crucial roles in reinforcing adsorption strength.

Compare the inhibition efficiency of the synthesized compound M with previous data on various chitosan derivatives presented in Table 7. The table highlights the remarkable inhibition efficiency of M at a concentration of 100 ppm. This demonstrates the potential of compound M as an effective and environmentally friendly inhibitor for carbon steel in acidic environments. In conclusion, chitosan M achieves inhibition efficiencies exceeding 90% at practical concentrations of 100 ppm establish it as a cost-effective inhibitor.

**Table 7:** Chitosan derivatives as corrosion inhibitor for CS in 1 M HCl comparison of the Inhibition efficiency (IE %) of our synthesized compound (M) with data from previous studies involving.

Inhibitor	Optimum concentration (ppm)	Corrosion tests	IE %	Ref.
Carboxymethyl chitosan butyraldehyde	250	PDP	82.9	^75^
*N*-vanillyl-*O*-2’-hydroxy propyl tri methyl ammonium chloride chitosan	200	WL, PDP, EIS	87.10	^76^
Chitosan-thiophene carbox aldehyde	1500	WL, PDP, EIS	73.60	^29^
Chitosan-benzaldehyde	50	WL, PDP, EIS	84.60	^77^
4-dimethylaminobenzaldehyde-chitosan	50	WL, PDP, EIS	87.30	^77^
Chitosan salicyladehyse	1500	PDP, EIS	90.50	^29^
Chitosan-p-toluene acid palmitic	250	WL, PDP, EIS	93.00	^78^
Polyaniline (PANI)/chitosan (CTS)	200	PDP, EIS	79.02	^79^
Chitosan Schiff Base	2000	WL, PDP, EIS	92.72	^80^.
Chitosan M	100	WL, OCP, PDP, EIS	99.80	Our work

### Quantum chemical calculations

**Fig. 16:**
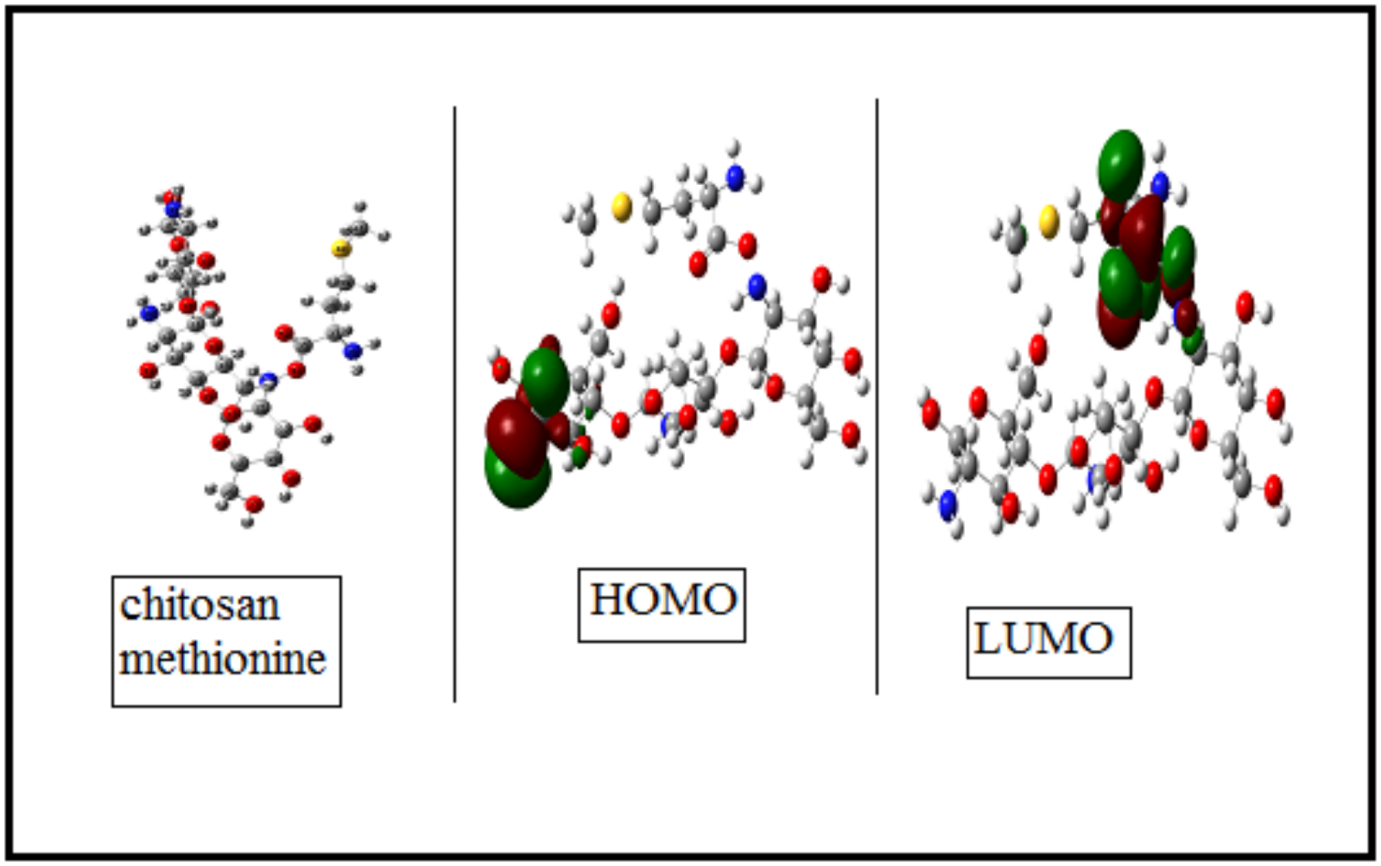
The molecular orbital of the synthesized compound M.

Quantum chemical computations are essential for examining the relationship between molecular structure of M and inhibition efficiency^81^. Furthermore, a theoretical analysis enables the pre-selection of substances possessing the necessary structural properties to act as effective organic corrosion inhibitors. Consequently, these theoretical approaches have been incorporated into numerous studies. The ideal shape for each synthesized compound showing the HOMO (highest occupied molecular orbital) and LUMO (lowest unoccupied molecular orbital) is shown in Fig. 16. The HOMO region is related to the compound’s tendency to donate electrons. Typically, the negatively charged heteroatom, π bonds, and phenyl rings constitute the HOMO regions and are therefore considered the most probable sites for adsorption. The LUMO regions represent the molecule’s tendency to accept electrons. In the diagram, the blue color represents Nitrogen, red represents Oxygen, grey represents Carbon and white represents Hydrogen.

Frontier orbital theory can be utilized to predict the adsorption centers of the inhibitor molecules that interact with the metal atoms. These centers include several key quantum chemical descriptors: *E*_LUMO_, *E*_HOMO_, the dipole moment (*µ*), the energy gap (Δ*E*_H–L_), the electron affinity (*A*), the ionization potential (*I*), the electronegativity (*χ)*, the chemical hardness (*η*), chemical softness (ξ), the chemical potential (µ), the maximum charge transfer index (∆N)and the electrophilicity index (*w*). However, it is important to understand that the molecular reactivity information derived from these quantum chemical descriptors cannot be directly translated into corrosion inhibition efficiency. The absorbability of an effective corrosion inhibitor is influenced by additional processes, such as film formation and surface characteristics. Therefore, a comparison of computed quantum chemical parameters and inhibition efficiency may not always result in a strong correlation.

It is well- known that a molecule’s capacity to donate electrons to acceptor molecules with low-energy, empty molecular orbital is correlated with its E_HOMO_ value. The higher E_HOMO_ value of molecule, the greater its tendency to donate electrons to these acceptor molecules. Increased E_HOMO_ values lead to enhanced adsorption of inhibitor molecules on the metal surface, thus increasing inhibition. This is accomplished by altering the adsorbed layer’s transport mechanism efficiency by modifying the transport mechanism of the adsorbed layer. Conversely, the molecule’s ability to accept electrons is indicated by the energy of the lowest unoccupied molecular orbital (*E*_LUMO_). A lower *E*_LUMO_ value suggests a higher likelihood of the molecule accepting electrons. Therefore, molecules with high *E*_HOMO_ value and low *E*_LUMO_ values are more effective as corrosion inhibitors due to their enhanced electron donating and electron accepting capability, respectively^82^.

The calculations listed in Table 8 showed that the highest energy E_HOMO_, which is expected to exhibit the highest corrosion inhibition. An essential factor in determining the inhibitor molecule’s responsiveness towards surface adsorption is the energy gap (*ΔE*_*H−L*_). A lower energy difference indicates that it will take less energy to remove an electron from the last occupied orbital, resulting in better inhibition efficiency. this lower energy gap facilitates the transfer of electrons between the inhibitor and the metal surface, enhancing the adsorption process and thereby improving the compound’s effectiveness as a corrosion inhibitor^83^.

It has been shown that organic compounds that can both donate electrons to and accept electrons from the metal’s empty orbital make good corrosion inhibitors. This dual capability enhances the inhibitor’s ability to interact with the metal surface, forming a protective layer that effectively reduces corrosion. The ability to donate electrons is associated with a high *E*_HOMO_ value, while the ability to accept electrons is linked to a low E_LUMO_ value. Together, these properties facilitate strong adsorption onto the metal surface, thereby enhancing the corrosion inhibition efficiency^84,85^. A molecule with a low energy gap is referred to as a “soft molecule” because it is more polarizable. Such molecules typically exhibit low kinetic stability and strong chemical activity. This increased polarizability allows them to easily interact with the metal surface, enhancing their effectiveness as corrosion inhibitors^86^.

**Table 8:** Some global reactivity descriptors the computed for M.

Parameters	Values for M	
E_HUMO_		− 0.209
E_LUMO_		− 0.022
Energy band gap (∆*E*)	∆*E* = *E*_HOMO_-*E*_LUMO_	− 0.187
Electron affinity (*A*)	*A*= − *E*_LUMO_	+ 0.022
Ionization potential (*I*)	*I*= − *E*_HOMO_	+ 0.209
Electronegativity (*χ*)	*χ* = (*I* + *A*)/2)	0.116
Chemical hardness (*η*)	*η* = (*I-A*)/2)	+ 0.093
Chemical softness *(ζ*)	*ζ* = 1/2*η*	5.34
Chemical potential (µ)	*µ* = − (*I + A)/*2	− 0.116
Electrophilicity index (*w*)	*w* = *µ*^2^ /2*η*	0.072
Maximum charge transfer index (Δ*N*_max_)	Δ*N*_max_ = − *µ*/*η*	1.24
Dipole moment		5.53

Regarding the ∆*E*_H–L_ parameter, it was observed that the lowest value, correlating with its superior inhibition efficiency. Another parameter that reflects the electrical distribution in molecules is the dipole moment, which can indicate whether a system is hydrophilic or hydrophobic. A high dipole moment suggests a polar molecule, while a low dipole moment suggests a nonpolar molecule. However it’s not always straight forward to establish a direct relationship between computed dipole moments and measured inhibition efficiency^44,87^.

Indeed, it’s widely accepted that better inhibition efficacy is expected from the adsorption of polar compounds with substantial dipole moments onto the metal surface. When quantum chemical calculations were compared with experimental inhibition efficiency, it was observed that the inhibitors demonstrated higher inhibition efficiencies as their dipole moment increased.

## Conclusions

The study presents the successful development and evaluation of a chitosan methionine derivative (M) as a promising eco-friendly corrosion inhibitor for carbon steel in a 1 M HCl solution, meeting the demand for sustainable solutions in industrial corrosion prevention. With notable inhibition efficiency, methionine demonstrates its effectiveness through various electrochemical tests, including PDP and EIS, indicated that the M compound acted as a mixed-type inhibitor. Its effectiveness increased with higher inhibitor concentrations. EIS results revealed the passivation of CS in the presence of M, evident from elevated *R*_p_ values. Inhibition efficiency rose with increasing inhibitor concentration. These outcomes suggest that the inhibitor operates through both surface adsorption onto the steel and the formation of a protective film, effectively reducing the corrosion rate.

The adsorption behavior of the chitosan derivative adhered closely to the Langmuir adsorption isotherm, indicating a robust and uniform interaction between the inhibitor molecules and the steel surface. The thermodynamic parameters calculated revealed the predominant physorption of M on the CS surface. M was adsorbed onto the carbon steel surface through the free electron pairs on N and S atoms.

In summary, the chitosan M emerges as a promising solution for safeguarding carbon steel in acidic conditions, offering high efficiency and cost-effectiveness. These findings represent a significant contribution to the realm of green chemistry and sustainable corrosion management, laying the groundwork for further advancements in eco-friendly corrosion inhibitors.

## Data Availability

All data generated or analyzed during this study are included in this published article.
